# Adenylyl Cyclase Type 8 Overexpression Impairs Phosphorylation-Dependent Orai1 Inactivation and Promotes Migration in MDA-MB-231 Breast Cancer Cells

**DOI:** 10.3390/cancers11111624

**Published:** 2019-10-23

**Authors:** Jose Sanchez-Collado, Jose J. Lopez, Isaac Jardin, Pedro J. Camello, Debora Falcon, Sergio Regodon, Gines M. Salido, Tarik Smani, Juan A. Rosado

**Affiliations:** 1 Department of Physiology, (Cellular Physiology Research Group), Institute of Molecular Pathology Biomarkers, University of Extremadura, 10003 Caceres, Spain; 2 Department of Physiology, (Smooth Muscle Physiology Research Group), Institute of Molecular Pathology Biomarkers, University of Extremadura, 10003 Caceres, Spain; 3 Department of Medical Physiology and Biophysics, Institute of Biomedicine of Sevilla, 41013 Sevilla, Spain

**Keywords:** orai1α, adenylyl cyclase 8, store-operated calcium entry, breast cancer cells, migration

## Abstract

Orai1 plays a major role in store-operated Ca^2+^ entry (SOCE) in triple-negative breast cancer (TNBC) cells. This channel is inactivated via different mechanisms, including protein kinase C (PKC) and protein kinase A (PKA)-dependent phosphorylation at Ser-27 and Ser-30 or Ser-34, respectively, which shapes the Ca^2+^ responses to agonists. The Ca^2+^ calmodulin-activated adenylyl cyclase type 8 (AC8) was reported to interact directly with Orai1, thus mediating a dynamic interplay between the Ca^2+^- and cyclic adenosine monophosphate (cAMP)-dependent signaling pathways. Here, we show that the breast cancer cell lines MCF7 and MDA-MB-231 exhibit enhanced expression of Orai1 and AC8 as compared to the non-tumoral breast epithelial MCF10A cell line. In these cells, AC8 interacts with the Orai1α variant in a manner that is not regulated by Orai1 phosphorylation. AC8 knockdown in MDA-MB-231 cells, using two different small interfering RNAs (siRNAs), attenuates thapsigargin (TG)-induced Ca^2+^ entry and also Ca^2+^ influx mediated by co-expression of Orai1 and the Orai1-activating small fragment (OASF) of STIM1 (stromal interaction molecule-1). Conversely, AC8 overexpression enhances SOCE, as well as Ca^2+^ entry, in cells co-expressing Orai1 and OASF. In MDA-MB-231 cells, we found that AC8 overexpression reduces the Orai1 phosphoserine content, thus suggesting that AC8 interferes with Orai1 serine phosphorylation, which takes place at residues located in the AC8-binding site. Consistent with this, the subset of Orai1 associated with AC8 in naïve MDA-MB-231 cells is not phosphorylated in serine residues in contrast to the AC8-independent Orai1 subset. AC8 expression knockdown attenuates migration of MCF7 and MDA-MB-231 cells, while this maneuver has no effect in the MCF10A cell line, which is likely attributed to the low expression of AC8 in these cells. We found that AC8 is required for FAK (focal adhesion kinase) phosphorylation in MDA-MB-231 cells, which might explain its role in cell migration. Finally, we found that AC8 is required for TNBC cell proliferation. These findings indicate that overexpression of AC8 in breast cancer MDA-MB-231 cells impairs the phosphorylation-dependent Orai1 inactivation, a mechanism that might support the enhanced ability of these cells to migrate.

## 1. Introduction

Breast cancer is one of the most common malignancies in women worldwide. Among the different subtypes, triple-negative breast cancer (TNBC) is more aggressive and exhibits a poorer prognosis than other types of breast cancer. Immunohistochemically, TNBC is characterized by the lack of estrogen and progesterone receptors or excess HER2 (human epidermal growth factor receptor 2) expression. Consequently, this type of cancer is resistant to hormonal therapies and chemicals that target the HER2 receptor [1]. Studies in TNBC cells revealed that Ca^2+^ signaling is remodeled and plays a key functional role [2,3,4,5]. TNBC cells overexpress Orai1, which is responsible for the activation of store-operated Ca^2+^ entry (SOCE) [6]. Orai1 is a well-characterized regulator of the proliferation and migration of many TNBC cells, including the MDA-MB-231 cell line [7,8,9].

Orai1 is the pore-forming subunit of the highly Ca^2+^-selective CRAC (Ca^2+^-release activated Ca^2+^) channel, the best characterized store-operated channel [10,11,12]. The CRAC channel is activated by the endoplasmic reticulum Ca^2+^ sensor STIM1 upon discharge of the intracellular Ca^2+^ stores, and the influx of Ca^2+^ through the channel is modulated by the regulation of STIM1 by proteins like SARAF (SOCE-associated regulatory factor) [13,14,15], as well as by CRAC channel inactivation. CRAC currents undergo Ca^2+^-dependent inactivation to prevent excessive Ca^2+^ influx. Two different mechanisms, termed fast Ca^2+^-dependent inactivation (FCDI) that occurs within milliseconds [16] and slow Ca^2+^-dependent inactivation (SCDI) that commences tens of seconds after Orai1 activation [17], were described, although the precise mechanism remains unclear. Two variants of Orai1, generated by alternative translation initiation, were recently identified [18], a long form termed Orai1α of approximately 33 kDa and a short form, Orai1β, lacking amino acids 1–63, of approximately 23 kDa. Orai1α exhibits a greater FCDI, thus suggesting that the N-terminal 63 amino acids might play a relevant role in this process [11]. A recent study reported direct interaction of Orai1 with the Ca^2+^ calmodulin-activated adenylyl cyclase type 8 (AC8) [19]. Willoughby et al. specifically identified interaction of the N-terminal region of AC8 with residues 26–34 of the Orai1 N-terminus [19]. This sequence, exclusively present in the Orai1α variant, overlaps with three Orai1 phosphorylation sites, Ser-27, -30, and -34. Ser-27 and -30 are phosphorylated by PKC in vivo and in vitro, leading to strong inactivation of CRAC channel function and SOCE [20]. On the other hand, a recent study demonstrated that AC8 mediates CRAC inactivation by phosphorylation of Orai1 at Ser-34 [21]. The functional interaction between AC8 and Orai1 reveals a finely regulated interplay between the cAMP and Ca^2+^ signaling pathways.

Here, we show that TNBC MDA-MB-231 cells overexpress Orai1 and AC8, with predominant overexpression of AC8 over Orai1. Interaction of AC8 with Orai1 interferes with phosphorylation of the latter, probably due to overlapping of the phosphorylation sites with the AC8-binding sequence of Orai1. In MDA-MB-231 cells, silencing of AC8 results in attenuation of SOCE, while AC8 overexpression enhances Ca^2+^ influx, thus suggesting that AC8 impairs the inactivation of Orai1. AC8 was also found to be required for breast cancer cell migration, thus suggesting that AC8, by enhancing cAMP levels and/or Ca^2+^ influx, plays an important functional role in breast cancer cells.

## 2. Results

### 2.1. Expression and Interaction of Orai1α and AC8 in Non-Tumoral and Breast Cancer Cell Lines

**Figure 1:**
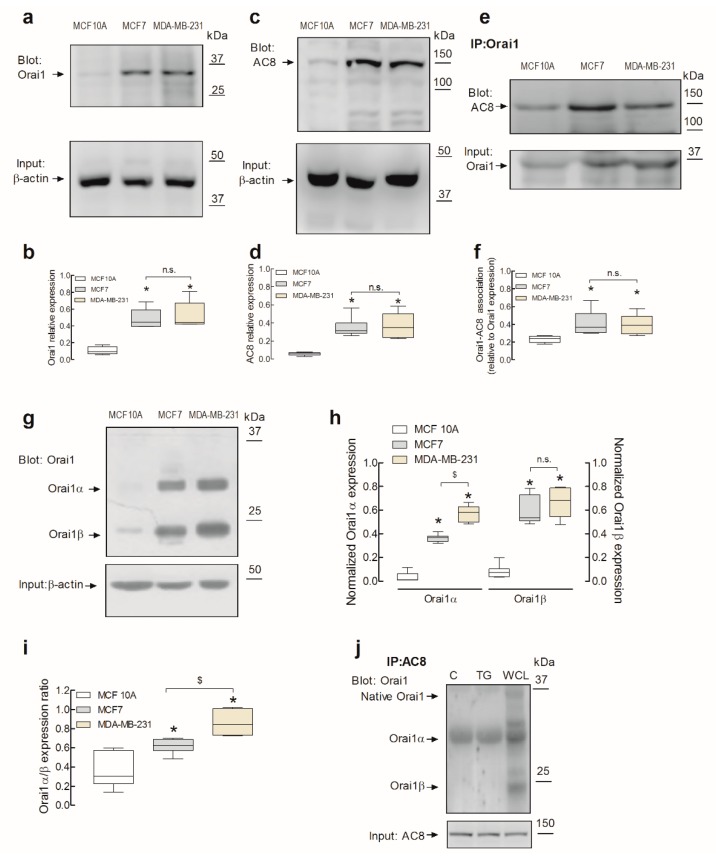
Expression and interaction of Orai1 variants with Ca^2+^ calmodulin-activated adenylyl cyclase type 8 (AC8) in non-tumoral and breast cancer cell lines. (**a**–**d**) Non-tumoral breast epithelial MCF10A and breast cancer MCF7 and MDA-MB-231 cells were lysed and subjected to Western blotting with anti-Orai1 (**a**) or anti-AC8 (**c**) antibody, followed by reprobing with anti-β-actin antibody for protein loading control (**b** and **d**). The box-and-whisker plots (or box plots) represent Orai1 (**b**) or AC8 (**d**) expression normalized to the β-actin content. Molecular masses indicated on the right were determined using molecular-mass markers run in the same gel; * *p* < 0.05 compared to the expression in MCF10A cells. (**e**) MCF10A, MCF7, and MDA-MB-231 cells were lysed, and whole-cell lysates were immunoprecipitated (IP) with anti-Orai1 antibody. Immunoprecipitates were subjected to 10% SDS-PAGE and subsequent Western blotting with specific anti-AC8 antibody, as indicated. Membranes were reprobed with the antibody used for immunoprecipitation for protein loading control. The panels show results from one experiment representative of five others. Molecular masses indicated on the right were determined using molecular-mass markers run in the same gel. (**f**) The box plot represents the quantification of AC8–Orai1 interaction in resting cells. Results are presented as arbitrary optical density units, and expressed normalized to the Orai1 expression. (**g**) MCF10A, MCF7, and MDA-MB-231 cells were lysed, and whole-cell lysates were treated with N-glycosidase F (PNGaseF) and resolved by 10% SDS-PAGE. The blots were probed with anti-Orai1 antibody and anti-β-actin antibody for loading control. Molecular masses indicated on the right were determined using molecular-mass markers run in the same gel. (**h**) The box plot represents Orai1α or Orai1β expression normalized to the β-actin content. (**i**) The box plot represents the Orai1α/Orai1β expression ratio in the three cell lines investigated; * *p* < 0.05 compared to the expression in MCF10A cells, ^$^ *p* < 0.05 compared to the expression in MCF7 cells. (**j**) MDA-MB-231 cells were treated with thapsigargin (TG; 1 µM) for 1 min or left untreated (C), as indicated, and lysed, and whole-cell lysates were immunoprecipitated (IP) with anti-AC8 antibody or subjected to Western blotting with anti-Orai1 antibody (WCL). Immunoprecipitates were treated with PNGaseF and then subjected to 10% SDS-PAGE and subsequent Western blotting with specific anti-Orai1 antibody, as indicated. Membranes were reprobed with the antibody used for immunoprecipitation for protein loading control. The panels show results from one experiment representative of five others. Molecular masses indicated on the right were determined using molecular-mass markers run in the same gel.

Consistent with previous studies [3,6], Western blot analysis of whole-cell lysates from the non-tumoral breast epithelial MCF10A cell line and the estrogen receptor positive (ER^+^) and TNBC cell lines MCF7 and MDA-MB-231, respectively, with a specific anti-human Orai1 antibody revealed a low expression of Orai1 in MCF10A cells and a significantly higher expression of this protein in breast cancer cells (Figure 1a,b; *p* < 0.05; *n* = 6). The increased expression of Orai1 in the breast cancer cell lines is consistent with the high expression of this protein in cancerous tissue [22]. As shown in Figure 1c,d, Western blot analysis of whole-cell lysates from MCF10A, MCF7, and MDA-MB-231 cells with a specific anti-AC8 antibody revealed that this protein is scarcely expressed in the non-tumoral cell line, while it is highly expressed in MCF7 and MDA-MB-231 breast cancer cells. The Orai1 and AC8 expression normalized to the β-actin content indicates that Orai1 expression was 371 ± 12 and 393 ± 22% of that in MCF10A cells in MCF7 and MDA-MB-231 cells, respectively, while the AC8 expression was 611 ± 75 and 621 ± 98% of that in MCF10A cells in MCF7 and MDA-MB-231 cells, respectively; therefore, the quantitative analysis indicated that AC8 overexpression in breast cancer cells is significantly greater than that of Orai1. Previous studies revealed a functional relationship between Orai1 and AC8 [19,21]; hence, we next explored the interaction between both proteins in the non-tumoral and tumoral breast cell lines by co-immunoprecipitation of cell lysates with anti-Orai1 antibody, followed by Western blotting with anti-AC8 antibody. The experiments were performed in resting cells as this interaction was previously shown to be constitutive [19]. Our results indicated that, while a detectable interaction was appreciated in non-tumoral cells, the co-immunoprecipitation between Orai1 and AC8 was significantly greater in MCF7 and MDA-MB-231 cells (Figure 1e,f; *p* < 0.05; *n* = 6). 

AC8 was reported to bind to an N-terminal sequence of Orai1 located between amino acids 26 and 34, which contains three serines (27, 30, and 34) [23]. This sequence is only present in the mammalian-specific full-length Orai1α variant and is absent in the short Orai1 variant, Orai1β [18]; thus, AC8 was reported to interact solely with Orai1α [21]. We assessed the expression of Orai1α and Orai1β in the three breast derived cell lines. The native Orai1 variant expression was analyzed by Western blotting after protein deglycosylation with PNGaseF. As shown in Figure 1g, two distinct bands with lower molecular weight than glycosylated Orai1 were detected, corresponding to Orai1α and Orai1β. Our results indicated that both Orai1 variants were highly expressed in the breast cancer MCF7 and MDA-MB-231 cell lines as compared to non-tumoral MCF10A cells (Figure 1h; *p* < 0.05; *n* = 6). Furthermore, we found that the expression of Orai1α was significantly greater in MDA-MB-231 cells than in MCF7 cells (Figure 1h; *p* < 0.05) while no differences were detected in the Orai1β expression among the cancer cell lines investigated. The latter might explain the greater Orai1α/Orai1β expression ratio in MDA-MB-231 cells as compared to MCF7 cells (Figure 1i; *p* < 0.05; *n* = 6). The analysis of the expression ratio between the Orai1 variants indicates a greater expression of Orai1β in all the cell types investigated and, interestingly, a greater Orai1α/Orai1β expression ratio in MCF7 and MDA-MB-231 breast cancer cells than in non-tumoral MCF10A cells (Figure 1i; *p* < 0.05; *n* = 6). We further explored the interaction of AC8 and Orai1α in MDA-MB-231 cells by co-immunoprecipitation of cell lysates with anti-AC8 antibody, followed by treatment of the immunoprecipitates with PNGaseF and Western blotting with anti-Orai1 antibody. As shown in Figure 1j, our results indicate that Orai1α, but not Orai1β, co-immunoprecipitates with AC8 in resting conditions, confirming previous results [19,21]. This interaction was not modified by treatment for 1 min with the sarco/endoplasmic reticulum Ca^2+^ ATPase (SERCA) inhibitor thapsigargin (TG; 1 µM), which is in agreement with a previous study by Willoughby and coworkers suggesting a constitutive interaction between both proteins [19].

### 2.2. The Interaction between Orai1 and AC8 Is Partially Dependent on Ca^2+^ Influx and Orai1 Phosphorylation at Ser-27 and -30

In order to explore the mechanism regulating the Orai1–AC8 interaction, we tested the possible Ca^2+^ dependency of this event. To address this issue, we assessed the role of Ca^2+^ released from the intracellular stores and Ca^2+^ entry through Orai1 in the Orai1–AC8 co-immunoprecipitation. Hence, we tested Orai1–AC8 interaction in resting cells or in cells treated with TG, to induce net Ca^2+^ release from intracellular Ca^2+^ stores, suspended either in the presence of 1 mM extracellular Ca^2+^ or in a Ca^2+^-free medium. When indicated, cells were loaded with dimethyl BAPTA, and suspended either in a Ca^2+^-free medium, to prevent rises in cytosolic Ca^2+^ concentration ([Ca^2+^]_c_) induced by both Ca^2+^ release and entry, or in the presence of 1 mM external Ca^2+^, to prevent rises in [Ca^2+^]_c_ due to Ca^2+^ release but allowing rises in Ca^2+^ concentration in the vicinity of the Orai1 channels.

**Figure 2:**
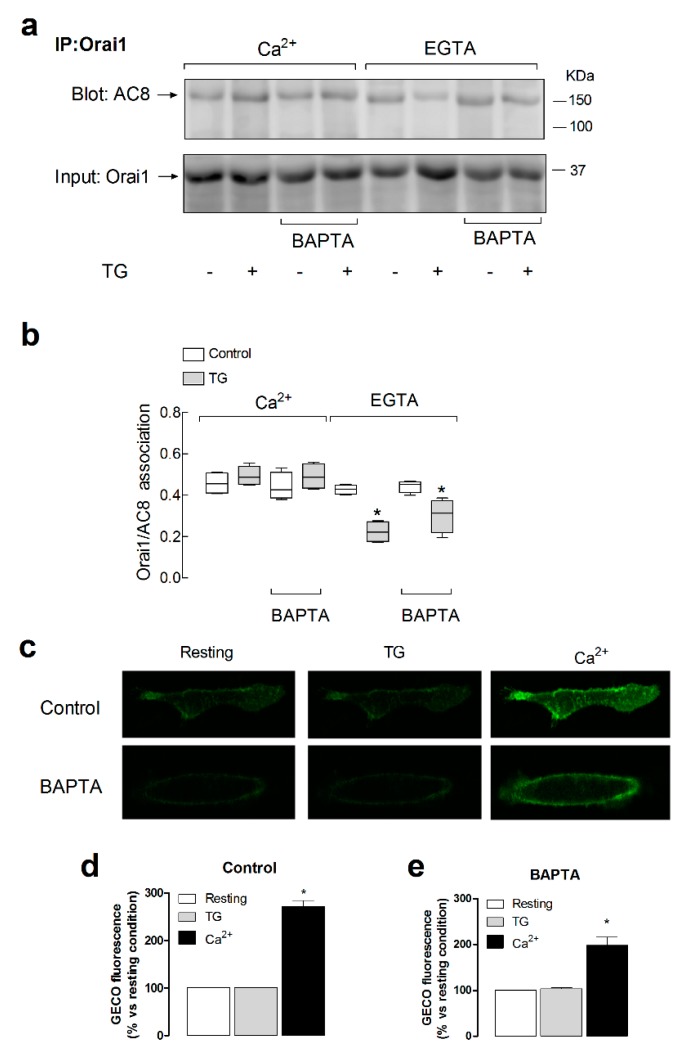
Role of Ca^2+^ mobilization in the Orai1–AC8 interaction. (**a**) MDA-MB-231 cells were loaded with dimethyl BAPTA or left untreated, as indicated, and then suspended in a medium containing 1 mM Ca^2+^ or in a Ca^2+^-free medium (100 µM EGTA added). Cells were treated with 1 µM TG or the vehicle, as indicated, and lysed 1 min later. Whole-cell lysates were immunoprecipitated (IP) with anti-Orai1 antibody. Immunoprecipitates were subjected to 10% SDS-PAGE and subsequent Western blotting with specific anti-AC8 antibody, as indicated. Membranes were reprobed with the antibody used for immunoprecipitation for protein loading control. The panels show results from one experiment representative of five others. Molecular masses indicated on the right were determined using molecular-mass markers run in the same gel. (**b**) The box plot represents the quantification of AC8–Orai1 interaction in resting and TG-treated cells. Results are normalized to the Orai1 expression; * *p* < 0.05 compared to the corresponding control (untreated cells). (**c**) Cells were transfected with G-GECO1.2-Orai1. Forty-eight hours later, cells were suspended in a Ca^2+^-free medium and stimulated with TG (1 µM) for 2 min, followed by addition of CaCl_2_ (final concentration 1 mM) to the medium to initiate Ca^2+^ entry. Images are representative of six independent experiments. (**d**–**e**) Bar graphs represent the quantification of the G-GECO (green genetically encoded Ca^2+^ indicator for optical imaging) fluorescence in cells loaded with dimethyl BAPTA (BAPTA) or left untreated (control), as indicated. Fluorescence was analyzed at rest, 30 s after the addition of 1 µM TG and 30 s after the subsequent addition of CaCl_2_ (final concentration 1 mM).

Our results indicated that, in the presence of extracellular Ca^2+^, there was a detectable Orai1–AC8 association, which was unaffected by BAPTA loading or treatment with TG (Figure 2a,b; *n* = 6), as previously reported [19]. Figure 2c–e depict a detectable increase in the Ca^2+^ concentration in the Orai1 vicinity in cells not loaded with BAPTA, as well as, with less intensity but still significant, in BAPTA-loaded cells as detected with G-GECO1.2-Orai1 [24] (*p* < 0.05; *n* = 6), and similar results were obtained using the near plasma membrane Ca^2+^ indicator fura-FFP18 (the initial slopes of the increase in fura-FFP18 fluorescence ratio were 1.0299 ± 0.1325 and 0.3793 ± 0.0205 in control and BAPTA-loaded cells, respectively, and the maximal fura-2 fluorescence ratios were 0.20 ± 0.01 and 0.11 ± 0.01 in control and BAPTA-loaded cells, respectively, Figure S1, Supplementary Materials), which demonstrate that detectable rises in Ca^2+^ concentration in the Orai1 microdomain due to Ca^2+^ influx via Orai1 were still detectable in BAPTA-loaded cells. Cell loading with dimethyl BAPTA was without a significant effect on the resting fura-FFP18 fluorescence ratio (the resting ratios were 0.60 ± 0.03 and 0.61 ± 0.01 in control and BAPTA-loaded cells, respectively). When Ca^2+^ entry was not allowed, treatment with TG resulted in a reduction in the Orai1–AC8 interaction, a response that was maintained when the rise in [Ca^2+^]_c_ due to Ca^2+^ efflux from the stores was prevented by BAPTA loading (Figure 2a,b; *n* = 6). These findings suggest that Ca^2+^ store depletion itself plays an inhibitory role in the Orai1–AC8 association that was overcome by Ca^2+^ influx via Orai1 channels. Therefore, upon agonist stimulation, the Orai1–AC8 interaction is strongly dependent on Ca^2+^ influx through the channel.

**Figure 3:**
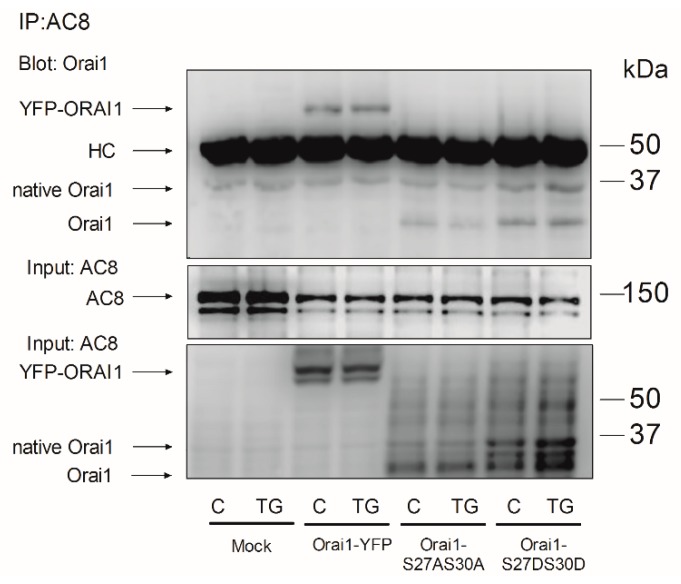
Role of Orai1 phosphorylation at Ser-27 and Ser-30 in the Orai1–AC8 interaction. MDA-MB-231 cells were transfected with pEYPF-Orai1, or the Orai1S27A/S30A and Orai1S27D/S30D mutants, or they were mock-treated, as indicated. Cells were then treated with 1 µM TG or the vehicle (C) and lysed 1 min later. Whole-cell lysates were immunoprecipitated (IP) with anti-AC8 antibody. Immunoprecipitates were subjected to 10% SDS-PAGE and subsequent Western blotting with specific anti-Orai1 antibody, as indicated. Membranes were reprobed with the anti-AC8 antibody for protein loading control. Alternatively, the cell lysates were subjected to 10% SDS-PAGE and subsequent Western blotting with anti-Orai1 antibody. The panels show results from one experiment representative of five others. Molecular masses indicated on the right were determined using molecular-mass markers run in the same gel. HC: heavy chain of the antibody used for immunoprecipitation; native Orai1: post-translationally modified Orai1; Orai1: Orai1 without post-translational modifications.

Orai1 is phosphorylated by PKC at residues Ser-27 and Ser-30, an event that negatively regulates Orai1 function [20]. As both serine residues are located within the Orai1 AC8-binding region, we explored whether phosphorylation at Ser-27 and Ser-30 alters Orai1–AC8 interaction. To investigate this issue, cells were transfected with yellow fluorescent protein (YFP)-Orai1, the non-phosphorylatable Orai1S27A/S30A mutant, or the phosphomimetic Orai1S27D/S30D mutant, or they were mock-treated, and the Orai1–AC8 interaction was analyzed by co-immunoprecipitation from cell lysates. Figure 3, bottom panel, depicts that expression of YFP-Orai1 produced a band of the predicted size (approximately 60 kDa). Furthermore, expression of the Orai1S27A/S30A and Orai1S27D/S30D mutants yielded several bands, one at the size of the native Orai1 and other small size bands which might be attributed to Orai1 without post-translational modifications. As shown in Figure 3, our results indicated that AC8 was able to co-immunoprecipitate with YFP-Orai1, as well as with the Orai1S27A/S30A and Orai1S27D/S30D mutants. Therefore, these findings indicate that phosphorylation of Orai1 at Ser-27 and Ser-30 is unlikely to interfere with Orai1 binding to AC8.

### 2.3. Role of AC8 in the Activation of Store-Operated Ca^2+^ Entry in Breast Cancer MDA-MB-231 Cells

**Figure 4:**
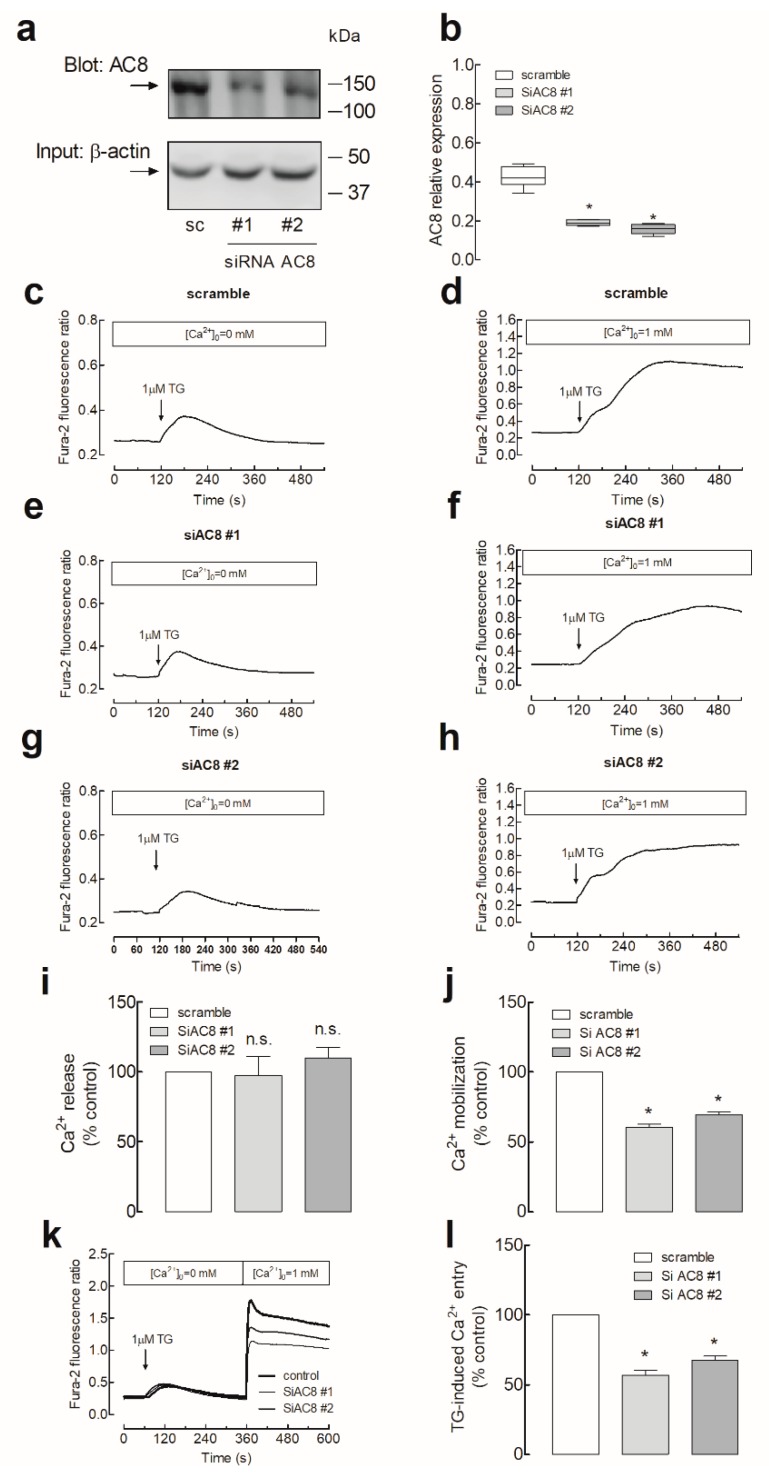
AC8 is required for full store-operated Ca^2+^ entry activation in MDA-MB-231 breast cancer cells. (**a**–**b**) MDA-MB-231 cells were transfected with two different small interfering RNA (siRNA) AC8 plasmids (siAC8#1 and siAC8#2) or a scramble plasmid (sc), as indicated. Forty-eight hours after transfection, cells were lysed and subjected to Western blotting with anti-AC8 antibody, followed by reprobing with anti-β-actin antibody for protein loading control (**a**). Molecular masses indicated on the right were determined using molecular-mass markers run in the same gel. (**b**) The box plot represents AC8 expression under the different experimental procedures normalized to the β-actin content. (**c**–**j**) MDA-MB-231 cells were transfected with siAC8#1, siAC8#2, or scramble plasmids, as indicated. Forty-eight hours after transfection, fura-2-loaded cells were perfused with a Ca^2+^-free medium (100 µM EGTA added) or with a medium containing 1 mM CaCl_2_, as indicated, and then stimulated with TG (1 µM). (**i** and **j**) Bar graphs represent TG-induced Ca^2+^ release (**i**) and mobilization (**j**) in MDA-MB-231 cells transfected with the indicated plasmids. Data are expressed as the AUC of means ± SEM (standard error of the mean) of 40 cells/day/3–5 days and presented as a percentage of control (cells transfected with scramble plasmid). (**k** and **l**) MDA-MB-231 cells were transfected with siAC8#1, siAC8#2, or scramble plasmids, as indicated. Forty-eight hours after transfection, fura-2-loaded cells were perfused with a Ca^2+^-free medium (100 µM EGTA added) and then stimulated with 1 µM TG, followed by addition of CaCl_2_ (final concentration 1 mM) to the medium to initiate Ca^2+^ influx. (**l**) Bar graphs represent TG-induced Ca^2+^ entry under the different experimental conditions. Data are expressed as the AUC of means ± SEM of 40 cells/day/3–5 days and presented as a percentage of control (cells transfected with scramble plasmid); * *p* < 0.05 as compared to scramble-treated cells.

SOCE in MDA-MB-231 cells was reported to be entirely dependent on Orai1 function [3,6]. Hence, we explored the functional role of the interaction between Orai1 and AC8 in these cells. To assess the role of AC8 in SOCE, MDA-MB-231 cells were transfected with two different commercial small interfering RNAs (siRNAs) for AC8 or scramble plasmids to analyze their effect on TG-evoked Ca^2+^ mobilization. As shown in Figure 4a,b, transfection with both plasmids attenuated the AC8 expression by about 50% in 48 h. As depicted in Figure 4c, in cells transfected with scramble plasmids suspended in a Ca^2+^-free medium, treatment with the SERCA inhibitor TG (1 µM) resulted in a transient increase in the fura-2 fluorescence ratio due to Ca^2+^ release from the intracellular Ca^2+^ stores. Cell stimulation with TG in a medium containing 1 mM Ca^2+^ resulted in a greater and sustained rise in the fura-2 fluorescence ratio as a result of Ca^2+^ release from the intracellular stores and entry through plasma membrane channels (Figure 4d). Cell transfection with the siAC8 plasmids was without significant effect on the resting fura-2 fluorescence ratio (in the absence of extracellular Ca^2+^, the resting ratios were 0.27 ± 0.01, 0.26 ± 0.01 and 0.28 ± 0.01 in cells transfected with scramble plasmid, siAC8#1 and siAC8#2, respectively, while, in the presence of 1 mM extracellular Ca^2+^, the resting ratios were 0.30 ± 0.02, 0.32 ± 0.01 and 0.33 ± 0.02 in cells transfected with scramble plasmid, siAC8#1 and siAC8#2, respectively). Furthermore, transfection with the siAC8 plasmids did not modify TG-induced Ca^2+^ release (Figure 4e,g,i). The initial peak fura-2 fluorescence ratios were 0.10 ± 0.01, 0.10 ± 0.01, and 0.09 ± 0.02 in cells transfected with scramble plasmid, siAC8#1, or siAC8#2, respectively, thus indicating that AC8 does not have a significant effect on the ability of MDA-MB-231 cells to accumulate Ca^2+^ in the intracellular stores or on the Ca^2+^ leakage rate from the stores. By contrast, attenuation of AC8 expression by transfection of the siAC8 plasmids reduced TG-evoked Ca^2+^ mobilization in the presence of 1 mM extracellular Ca^2+^ (Figure 4f,h,i; the initial slopes of the increase in fura-2 fluorescence ratio were 0.0065 ± 0.0004, 0.0036 ± 0.0003, and 0.0040 ± 0.0002 in cells transfected with scramble plasmid, siAC8#1, or siAC8#2, respectively, while the initial peak fura-2 fluorescence ratios were 0.93 ± 0.02, 0.64 ± 0.03, and 0.65 ± 0.02, in cells transfected with scramble plasmid, siAC8#1, or siAC8#2, respectively). As TG-evoked Ca^2+^ release was unaffected by attenuation of the AC8 expression, this effect should be attributed to a reduction in TG-induced Ca^2+^ influx. If we consider the area under the curve (AUC) or the entry of Ca^2+^ stimulated by TG, corrected by subtraction of the response to TG in the absence of external Ca^2+^, transfection of the siAC8#1 and siAC8#2 plasmids significantly reduced SOCE by 40 ± 2% and 31 ± 2%, respectively (Figure 4j; *p* < 0.05).

Similar results were observed when we estimated TG-induced Ca^2+^ entry using the Ca^2+^ add-back protocol. As shown in Figure 4k, transfection of siAC8#1 or #2 plasmids significantly attenuated SOCE (the initial peak fura-2 fluorescence ratios upon addition of Ca^2+^ to TG-treated cells were 1.68 ± 0.35, 0.90 ± 0.05, and 1.09 ± 0.04 in cells transfected with scramble plasmid, siAC8#1, or siAC8#2, respectively, and the initial slopes of the increase in fura-2 fluorescence ratio were 0.0448 ± 0.0091, 0.0213 ± 0.0022, and 0.0207 ± 0.0029 in cells transfected with scramble plasmid, siAC8#1, or siAC8#2, respectively).

**Figure 5:**
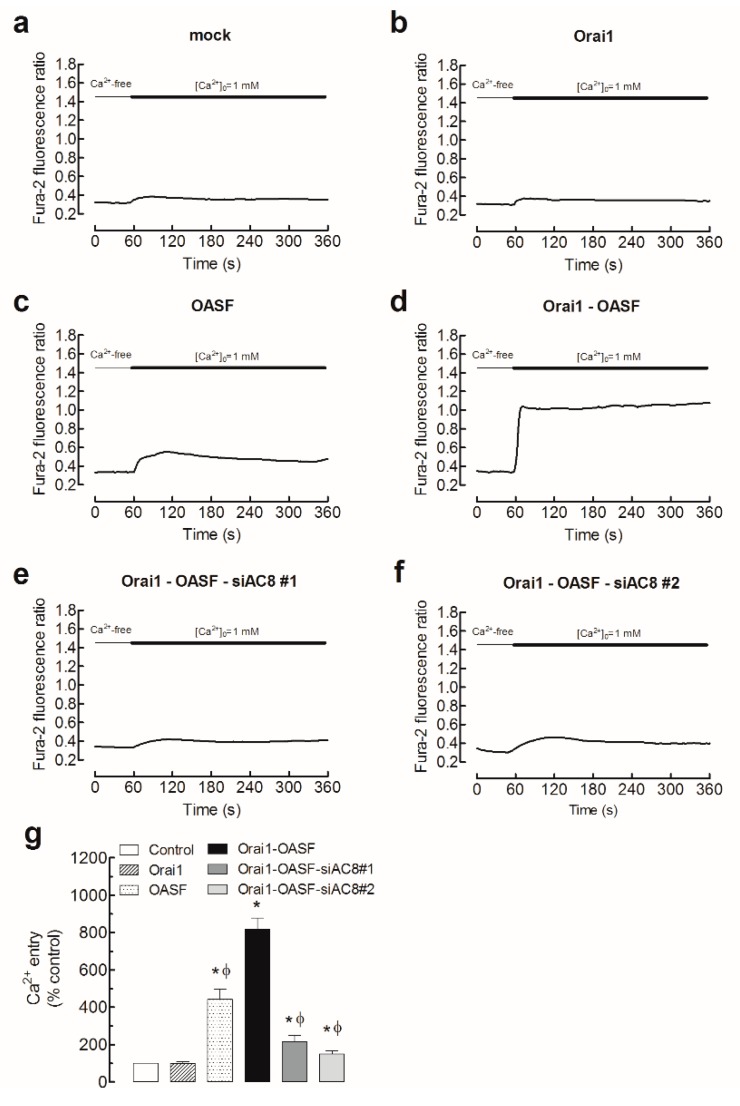
AC8 silencing attenuates Ca^2+^ influx mediated by Orai1-activating small fragment (Orai1-OASF) in MDA-MB-231 breast cancer cells. MDA-MB-231 cells were transfected with Orai1 (**b**), OASF (**c**), both transfection plasmids (**d**–**f**), or empty vector (mock, **a**), as indicated. Cells were also transfected with siAC8#1 (**e**), siAC8#2 (**f**), or scramble plasmid (**d**). Forty-eight hours after transfection, fura-2-loaded cells were perfused with a Ca^2+^-free medium (100 µM EGTA added), followed by reintroduction of external Ca^2+^ (final concentration 1 mM) to initiate Ca^2+^ entry. (**g**) Bar graphs represent Ca^2+^ entry in MDA-MB-231 cells transfected with the indicated plasmids. Data are expressed as means ± SEM of 40 cells/day/3–5 days and presented as a percentage of control cells; * *p* < 0.05 as compared to control, ^ϕ^ *p* < 0.05 as compared to cells transfected with Orai1 and OASF expression plasmids and scramble RNA.

In an attempt to ascertain more specifically the role of AC8 in Ca^2+^ influx through Orai1, we analyzed the entry of Ca^2+^ in cells expressing Orai1 and the Orai1-activating small fragment (OASF; amino acids 233–474) of STIM1. MDA-MB-231 cells were transfected with expression plasmids for pEYFP-Orai1 and pEYFP-OASF in combination with the siAC8 or scramble plasmids. Expression of Orai1, OASF, or both in the absence or presence of the siAC8 plasmids did not significantly alter the resting fura-2 fluorescence ratio (resting ratios were 0.30 ± 0.01, 0.32 ± 0.01, 0.30 ± 0.01, 0.32 ± 0.01, 0.30 ± 0.01, and 0.31 ± 0.01 in cells transfected with scramble plasmid, Orai1, OASF, Orai1 + OASF, Orai1 + OASF + siAC8#1, and Orai1 + OASF + siAC8#2, respectively). As shown in Figure 5, expression of Orai1 alone had a negligible effect, if any, on the fura-2 fluorescence ratio, as previously reported [25]. On the other hand, OASF expression significantly enhanced Ca^2+^ influx in these cells, which expressed a significant amount of endogenous Orai1 (Figure 5c). Co-expression of Orai1 and OASF resulted in a robust activation of Ca^2+^ entry independently of Ca^2+^ store depletion as compared to mock-treated cells, which did not display a significant constitutive Ca^2+^ entry. Ca^2+^ entry induced by co-expression of Orai1 and OASF was impaired by AC8 silencing (Figure 5e,f,g; *p* < 0.05), thus suggesting that AC8 directly modulates Ca^2+^ entry through Orai1 in MDA-MB-231 cells. The initial peak fura-2 fluorescence ratios were 0.09 ± 0.01, 0.10 ± 0.01, 0.28 ± 0.02, 0.84 ± 0.07, 0.13 ± 0.01, and 0.13 ± 0.01 in cells transfected with scramble plasmid, Orai1, OASF, Orai1 + OASF, Orai1 + OASF + siAC8#1, and Orai1 + OASF + siAC8#2, respectively.

**Figure 6:**
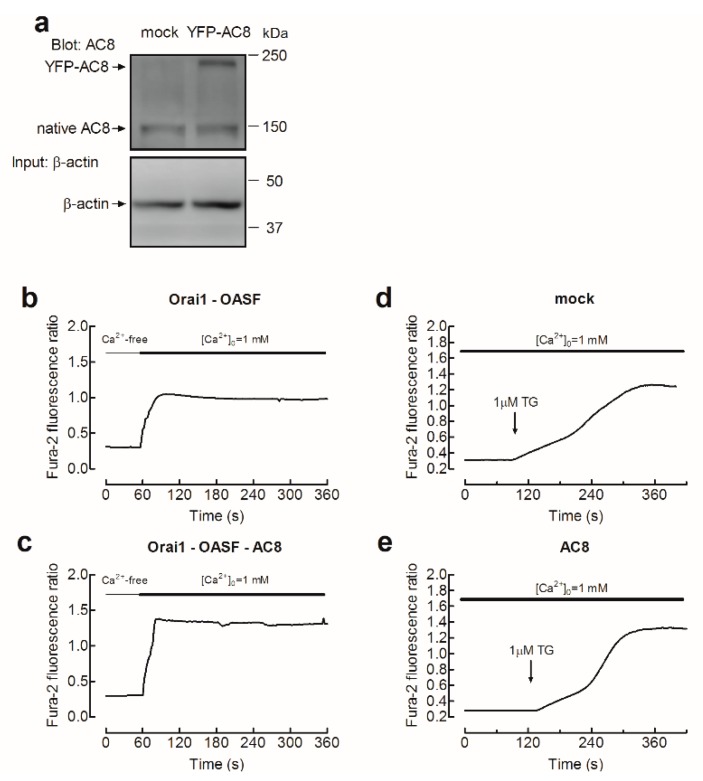
AC8 overexpression enhances store-operated Ca^2+^ influx in MDA-MB-231 breast cancer cells. (**a**) MDA-MB-231 cells were transfected with YFP-AC8 transfection plasmid or empty vector (mock), as indicated. Forty-eight hours after transfection, cells were lysed and subjected to Western blotting with anti-AC8 antibody, followed by reprobing with anti-β-actin antibody for protein loading control. Molecular masses indicated on the right were determined using molecular-mass markers run in the same gel. (**b**,**c**) Cells were transfected with Orai1 and OASF plasmids, as well as either with YFP-AC8 plasmid (**c**) or empty vector (**b**), as indicated. Forty-eight hours after transfection, fura-2-loaded cells were perfused with a Ca^2+^-free medium (100 µM EGTA added), followed by reintroduction of external Ca^2+^ (final concentration 1 mM) to initiate Ca^2+^ entry. (**d**,**e**) Cells were transfected with YFP-AC8 expression plasmid (**e**) or empty vector (mock, **b**), as indicated. Forty-eight hours after transfection, fura-2-loaded cells were perfused with a medium containing 1 mM CaCl_2_, as indicated, and then stimulated with TG (1 µM). Traces are representative of 40 cells/day/3–5 days.

The role of AC8 in Orai1 channel function in MDA-MB-231 cells was further explored by testing the effect of AC8 overexpression on Ca^2+^ influx in cells expressing exogenous Orai1 and OASF. Cell transfection with AC8 plasmid was without significant effect on the resting fura-2 fluorescence ratio (in the absence of extracellular Ca^2+^, the resting ratios were 0.34 ± 0.01, 0.36 ± 0.01, 0.32 ± 0.01, and 0.32 ± 0.01 in cells transfected with Orai1 + OASF, Orai1 + OASF + AC8, empty vector (mock), and AC8 overexpression plasmid alone, respectively; Figure 6). As shown in Figure 6a, YFP-AC8 was efficiently expressed in MDA-MB-231 cells. Interestingly, Ca^2+^ entry induced by co-expression of Orai1 and OASF, estimated as the AUC, was significantly enhanced by 30% upon AC8 overexpression (Figure 6b,c; *p* < 0.05). The initial peak fura-2 fluorescence ratios were 0.84 ± 0.02, 1.01 ± 0.03, 0.89 ± 0.04, and 1.11 ± 0.05 in cells transfected with Orai1 + OASF, Orai1 + OASF + AC8, empty vector (mock), and AC8 overexpression plasmid alone, respectively, whereby the two latter ones occurred upon stimulation with TG (see Figure 6d,e). Furthermore, the initial slope of the increase in fura-2 fluorescence ratio upon stimulation with TG in the presence of 1 mM extracellular Ca^2+^ was also enhanced by AC8 overexpression from 0.0062 ± 0.0003 to 0.0080 ± 0.0003 in mock-treated and AC8-transfected cells, respectively (Figure 6d,e). These findings further suggest that AC8 plays a positive role in Orai1 channel function in MDA-MB-231 cells. Accordingly, Ca^2+^ mobilization evoked by TG in the presence of 1 mM extracellular Ca^2+^ was enhanced.

### 2.4. AC8 Prevents Phosphorylation of Orai1 at Ser-27 and -30

**Figure 7:**
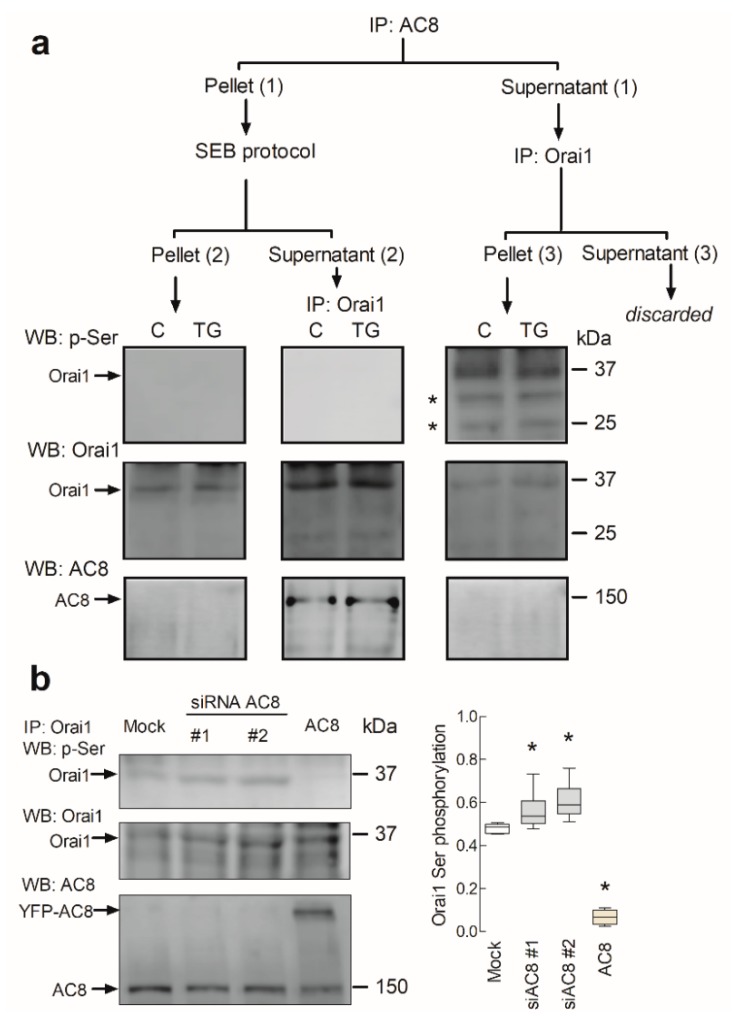
AC8 modulates Orai1 serine phosphorylation. (**a**) Phosphorylation of AC8-associated and -independent Orai1 at Ser-27 and -30. MDA-MB-231 cells were stimulated with 1 µM TG or the vehicle (C) and lysed 1 min later. Whole-cell lysates were immunoprecipitated (IP) with anti-AC8 antibody, yielding pellet (1) and supernatant (1). The proteins of pellet (1) were eluted with soft elution buffer (SEB). This step resulted in pellet (2) and supernatant (2). Pellet (2) was subjected to 10% SDS-PAGE and subsequent Western blotting with anti-phosphoserine antibody. Supernatant (2) was immunoprecipitated with anti-Orai1 antibody, followed by Western blotting with anti-phosphoserine antibody. On the other hand, supernatant (1) was immunoprecipitated with anti-Orai1 antibody, leading to pellet (3) and supernatant (3) that was discarded. Pellet (3) was subjected to 10% SDS-PAGE and subsequent Western blotting with specific anti-phosphoserine antibody, as indicated. Membranes were reprobed with anti-Orai1 and anti-AC8 antibodies for protein loading control. The panels show results from one experiment representative of five others. Molecular masses indicated on the right were determined using molecular-mass markers run in the same gel; * nonspecific bands. (**b**) MDA-MB-231 cells were transfected with YFP-AC8 (AC8) transfection plasmid, siAC8#1, siAC8#2 or empty vector (Mock), as indicated. Forty-eight hours after transfection, cells were lysed. Whole-cell lysates were immunoprecipitated (IP) with anti-Orai1 antibody. Immunoprecipitates were subjected to 10% SDS-PAGE and subsequent Western blotting with specific anti-phosphoserine antibody, as indicated. Membranes were reprobed with the anti-Orai1 antibody for protein loading control and anti-AC8. The panels show results from one experiment representative of four others. Molecular masses indicated on the right were determined using molecular-mass markers run in the same gel. The box plot represents the Orai1 phosphoserine content relative to the Orai1 expression.

We further explored the mechanism involved in the regulation of Orai1 channel function by AC8. A recent study reported that Ca^2+^ entry via Orai1 results in the activation of AC8, which, in turn, generates cAMP and activates PKA. The latter phosphorylates Orai1 at Ser-34, leading to Ca^2+^-dependent inactivation of the channel [21]. Furthermore, Orai1 phosphorylation at Ser-27 and -30 is associated with suppression of Orai1 channel function [20]. As phosphorylation of Ser-27, -30, and -34 is associated with Orai1 inactivation, and as these residues are located within the Orai1 AC8-binding sequence, we further evaluated whether interaction with AC8 prevents phosphorylation of Orai1 at serine residues. To investigate this issue, we assessed the phosphoserine content of the AC8-associated and AC8-independent Orai1 subsets in MDA-MB-231 cells. Resting cells and cells stimulated with TG were used to elucidate the involvement of Ca^2+^ store depletion in Orai1 Ser-27 and Ser-30 phosphorylation. To analyze Orai1 phosphorylation in the AC8-associated and independent Orai1 subsets, cell lysates were immunoprecipitated with anti-AC8 antibody, and the supernatants (AC8-independent Orai1 fraction), as well as the proteins eluted from the pellet (AC8-bound Orai1), were immunoprecipitated again with anti-Orai1 antibody, followed by Western blotting with anti-phosphoserine antibody. Our results showed a detectable phosphorylation of the AC8-independent Orai1 fraction at serine residues in resting cells, which was maintained upon treatment with TG (Figure 7, pellet (3); *n* = 6). By contrast, we were unable to detect serine phosphorylation in the AC8-associated Orai1 fraction (Figure 7, supernatant (2); *n* = 6). As a control, we tested the phosphoserine content of the proteins remaining in the AC8 immunoprecipitates after the elution with soft elution buffer (SEB); however, although some Orai1 was still remaining in the sample, Western blotting with the specific anti-phosphoserine antibody was unable to detect any bands. Therefore, these findings indicate that interaction with AC8 prevents Orai1 serine phosphorylation, which might impair inactivation of the channel.

To further explore the modulation of Orai1 phosphorylation at serine residues by AC8, we tested the effect of attenuation of AC8 expression and overexpression of the Orai1 phosphoserine content. As shown in Figure 7b, cell transfection with siAC8#1 or siAC8#2 slightly enhanced Orai1 phosphorylation at serine residues, while, more interestingly, AC8 overexpression almost completely abolished Orai1 phosphorylation (*p* < 0.05; *n* = 5). These findings provide evidence for a role of AC8 in the regulation of Orai1 phosphorylation.

### 2.5. AC8 Plays a Relevant Role in MDA-MB-231 Cell Migration

**Figure 8:**
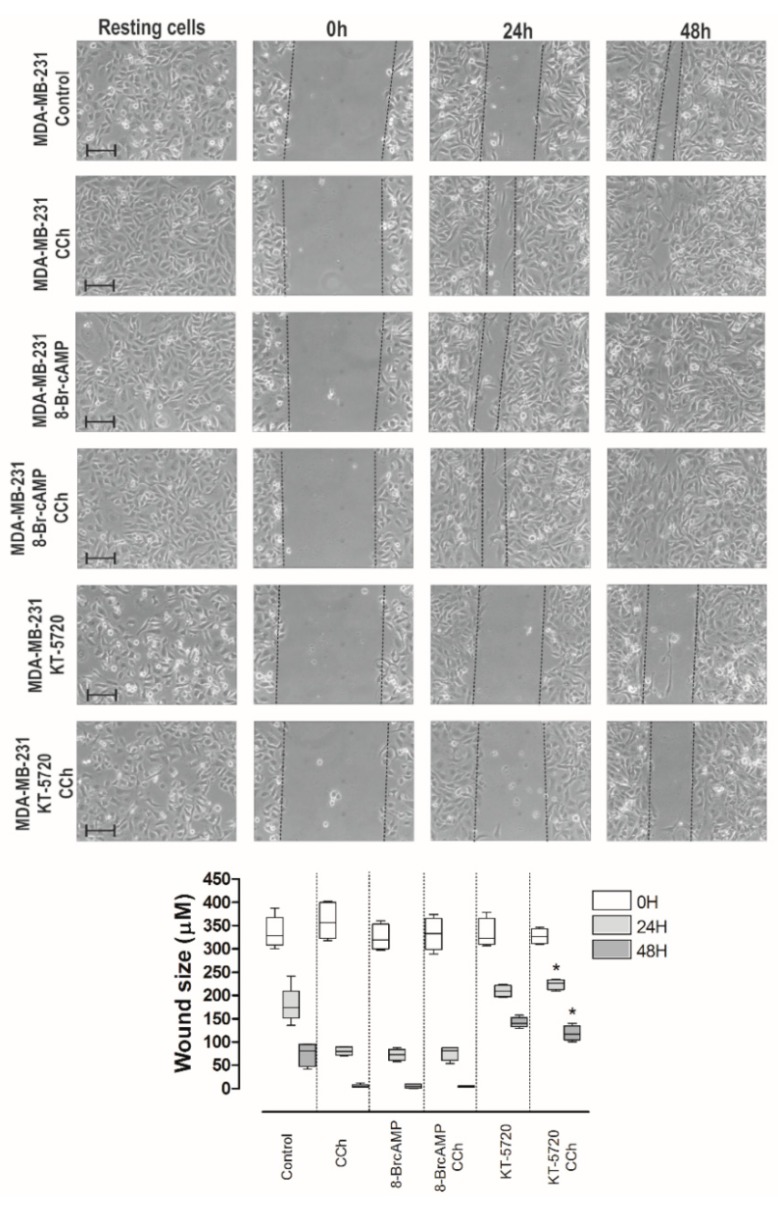
Role of the cAMP–PKA pathway in MDA-MB-231 cell migration. MDA-MB-231 cells were stimulated with 10 µM carbachol (CCh) or the vehicle in the absence or presence of 8-bromo-cAMP (8-Br-cAMP; 300 µM) or KT-5720 (1 µM), and subjected to a wound healing assay as described in Section 4. Images were acquired at 0, 24, and 48 h from the beginning of the assay. The dotted lines define the areas lacking cells. The bars represent 100 µm. The box plot represents the wound size, in micrometers, at the different conditions (*n* = 6); * *p* < 0.05 compared to the corresponding time in CCh-treated cells.

Ca^2+^ influx via Orai1 channels was reported to play a relevant role in the development of a number of cancer hallmarks, including cell migration [3,26,27,28]. Next, we assessed the relevance of AC8 in the ability of these cell lines to migrate. AC8 is a point of convergence between Ca^2+^ and cAMP; therefore, we firstly explored the effect of increasing cAMP levels in MDA-MB-231 cell migration using the well-established wound healing assay. Cells were seeded, scratched, and cultured in medium supplemented with 1% serum to prevent further cell growth. Migration of cells was quantitated as described in Section 4. As shown in Figure 8, MDA-MB-231 cells reduced the wound size during the first 48 h, and cell migration was enhanced by stimulation of MDA-MB-231 cells with the cholinergic agonist carbachol (CCh; 10 µM). Cell treatment with the cell permeant cAMP analogue and PKA activator [29], 8-bromo-cAMP (8-Br-cAMP; 300 µM), enhanced the ability of MDA-MB-231 cells to migrate to a similar extent as CCh, so that stimulation with CCh in the presence of 8-Br-cAMP did not increase the effect of 8-Br-cAMP alone (Figure 8; *n* = 6). To further explore the role of PKA in MDA-MB-231 cell migration, cells were pretreated for 30 min with the PKA inhibitor KT-5720 (1 µM) before they were scratched. Consistent with the results obtained with 8-Br-cAMP, treatment with KT-5720 significantly attenuated migration of CCh-stimulated and untreated MDA-MB-231s (Figure 8; *p* < 0.05; *n* = 6). Similar results were observed when we tested the effect of 8-Br-cAMP and KT-5720 on MCF7 cell migration (Figure S2, Supplementary Materials). Altogether, these findings indicate that the cAMP–PKA pathway plays an important role in luminal MCF7 and triple-negative MDA-MB-231 breast cancer cell migration.

**Figure 9:**
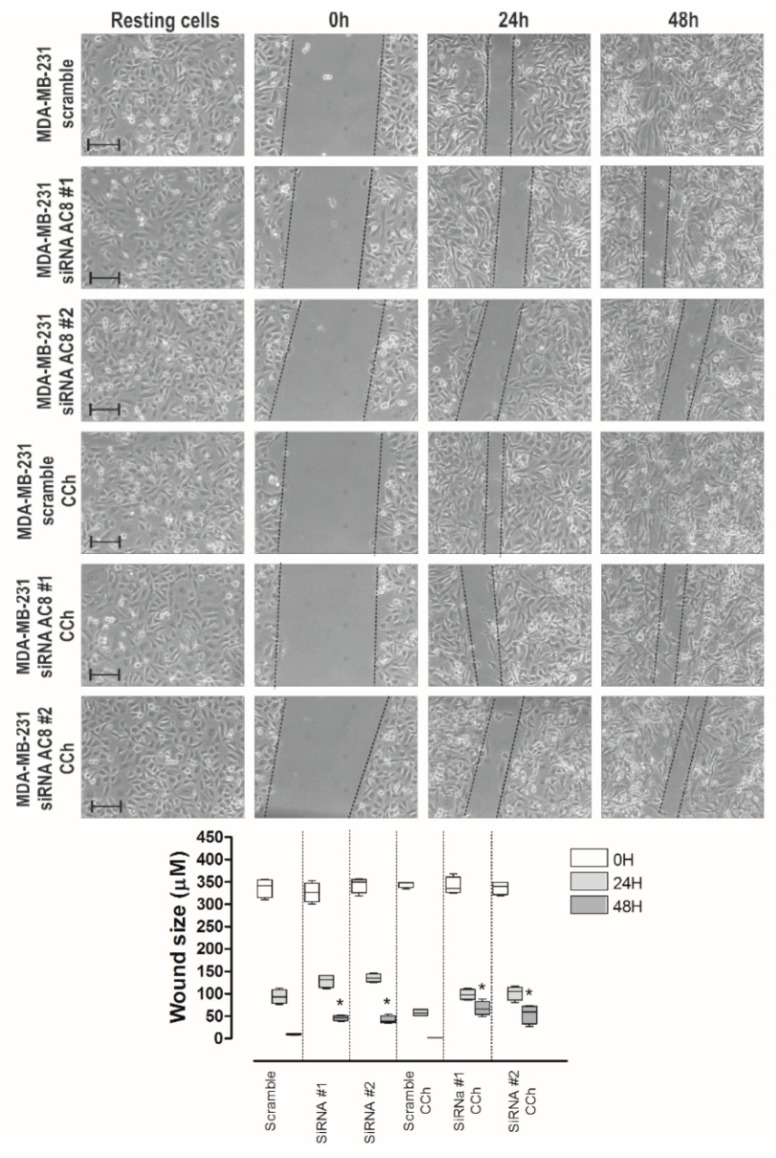
Role of AC8 in MDA-MB-231 cell migration. MDA-MB-231 cells were transfected with siAC8#1, siAC8#2, or scramble plasmid, as indicated. Forty-eight hours after transfection, cells were stimulated with 10 µM CCh or the vehicle and subjected to a wound healing assay as described in Section 4. Images were acquired at 0, 24, and 48 h from the beginning of the assay. The dotted lines define the areas lacking cells. The bars represent 100 µm. The box plot represents the wound size, in micrometers, at the different conditions (*n* = 6); * *p* < 0.05 compared to the corresponding time in cells transfected with scramble plasmid.

Next, we more specifically explored the relevance of AC8 in the ability of MDA-MB-231 cells to migrate. MDA-MB-231 cells were transfected with siAC8#1 and siAC8#2 or scramble plasmids, and cell migration was evaluated. As shown in Figure 9, MDA-MB-231 cells transfected with scramble plasmid significantly reduced the wound size during the first 48 h. AC8 expression attenuation significantly attenuated MDA-MB-231 cell migration as compared to cells transfected with scramble plasmid (Figure 9; *p* < 0.05; *n* = 6). Similarly, transfection of siAC8#1 and siAC8#2 plasmids significantly attenuated cell migration stimulated by CCh. These findings indicate that AC8 plays an important role in MDA-MB-231 cell migration. As depicted in Figure S3 (Supplementary Materials), attenuation of AC8 expression in MCF7 by transfection of siAC8#1 and siAC8#2 plasmids significantly reduced migration whether in non-stimulated or CCh-stimulated cells (*n* = 6). By contrast, AC8 expression silencing did not affect the ability of non-tumoral breast epithelial MCF10A cells to migrate (Figure S4, Supplementary Materials; *n* = 6), which is consistent with the low expression of TRPC6 (canonical transient receptor potential channel-6) in this cell line (see Figure 1c). These findings indicate that AC8 plays an important role in cell migration specifically in breast cancer cells.

**Figure 10:**
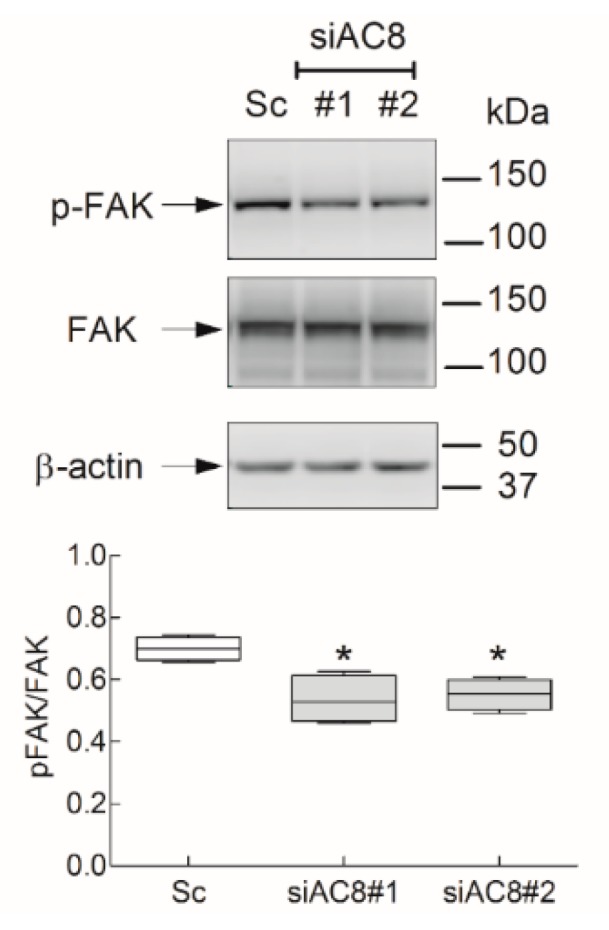
Role of AC8 in focal adhesion kinase (FAK) tyrosine phosphorylation in MDA-MB-231 cells. MDA-MB-231 cells were transfected with siAC8#1, siAC8#2, or scramble plasmid (Sc), as indicated. Forty-eight hours later, cells were lysed and subjected to 10% SDS-PAGE and Western blotting with anti-phospho-FAK (Y^397^) or anti-FAK specific antibodies. Membranes were reprobed with anti-β-actin antibody for protein loading control. Blots are representative of five separate experiments. The box plot represents FAK tyrosine phosphorylation presented as the phospho-FAK/total FAK ratio.

Phosphorylation and activation of the focal adhesion kinase (FAK) was shown to play an important role in cell migration and invasion [30]. FAK is a 125-kDa tyrosine kinase associated with focal adhesions [31], and phosphorylation at Tyr-397 is generally accepted as the initial step in FAK activation [32]. We previously found that Orai1 is involved in FAK phosphorylation in cancer cells [27]; therefore, we investigated the role of AC8 in FAK phosphorylation in MDA-MB-231 cells. As illustrated in Figure 10, AC8 expression attenuation by transfection of siAC8 plasmids significantly inhibited FAK phosphorylation at Tyr-397 as compared to cells transfected with scramble plasmid. These observations indicate that AC8 is required for full tyrosine phosphorylation and activation of FAK, which might underlie its role in MDA-MB-231 cell migration.

### 2.6. AC8 Is Required for TNBC Cell Proliferation

**Figure 11:**
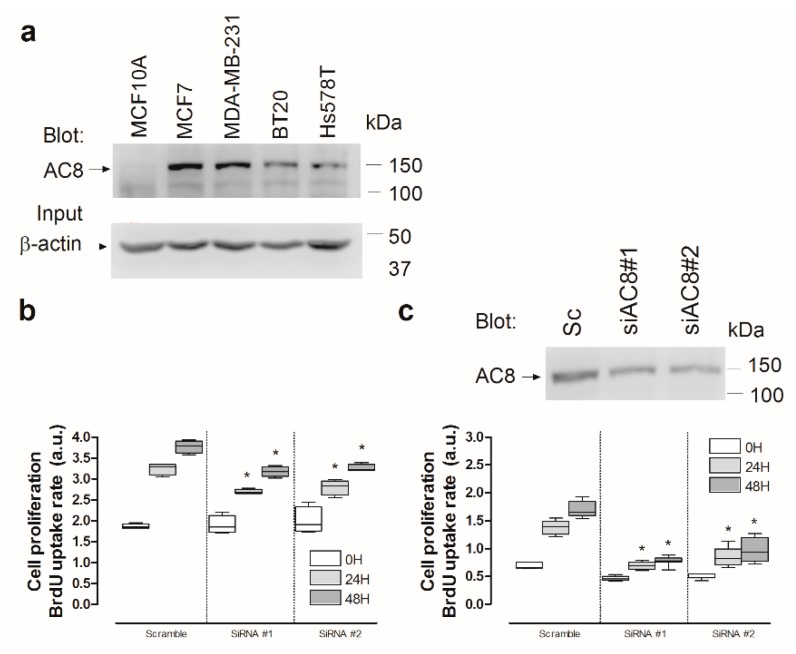
Role of AC8 in triple-negative breast cancer (TNBC) cell proliferation. (a) Non-tumoral breast epithelial MCF10A, luminal MCF7 breast cancer, and TNBC cells (MDA-MB-231, BT20, and Hs578T) were lysed and subjected to Western blotting with anti-AC8 antibody, followed by reprobing with anti-β-actin antibody for protein loading control. (b,c) MDA-MB-231 (b) and Hs578T (c) cells were transfected with siAC8#1, siAC8#2, or scramble plasmid (Sc), as indicated. Forty-eight hours later, cell proliferation was assessed for a further 24 and 48 h using the bromodeoxyuridine (BrdU) cell proliferation assay kit, as described in Section 4. The box plot represents cell proliferation 0, 24, and 48 h after cell transfection, presented as BrdU uptake rate; * *p* < 0.05 compared to the corresponding control (cells transfected with scramble plasmid). (c, top panel) Hs578T cells were lysed and subjected to 10% SDS-PAGE and Western blotting with anti-AC8 antibody. Blots are representative of three separate experiments.

We further explored the role of AC8 in cell proliferation in TNBC cells. Western blot analysis of whole-cell lysates from MCF10A and MCF7, as well as the TNBC cell lines MDA-MB-231, BT20, and Hs578T, with a specific anti-AC8 antibody revealed that this protein is highly expressed in MCF7 and TNBC cells (Figure 11a). Next, we explored the role of AC8 in MDA-MB-231 and HS578T cells. As shown in Figure 4a and 11c, cell transfection with siAC8#1 and siAC8#2 significantly attenuated AC8 expression in MDA-MB-231 and Hs578T cells, respectively (*p* < 0.05; *n* = 4). Silencing AC8 protein expression significantly attenuated MDA-MB-231 and Hs578T cell proliferation at all times investigated as compared to cells transfected with scramble plasmid (Figure 11b,c; *p* < 0.05; *n* = 4). Therefore, our observations reveal that AC8 plays an important role in TNBC cell proliferation.

## 3. Discussion

Orai1 was reported to play a major role in Ca^2+^ influx in the TNBC cell line MDA-MB-231. SOCE, a major mechanism for Ca^2+^ entry in this cell type, was found to be strongly dependent on Orai1 [6], whose plasma membrane expression is modulated by TRPC6 channels [3]. Furthermore, Orai1 is involved in store-independent Ca^2+^ influx through a functional interaction with Kv10.1 potassium channels that mediate serum-induced migration [9]. Orai1 Ca^2+^ channels play an important functional role in breast cancer cells supporting a number of cancer hallmarks such as migration, proliferation, or survival [33,34,35].

The Orai1 N-terminal intracellular region contains several phosphorylation sites. Ser-27 and -30 are the main phosphorylation sites targeted by PKCβ1 [20], while Ser-34 was found to be a target of protein kinase G (PKG) [36] and, more recently, Zhang and coworkers revealed that Ser-34 is also a PKA phosphorylation site [21]. Phosphorylation of Orai1 at any of these serine residues was demonstrated to result in Orai1 channel inactivation and reduction of Ca^2+^ influx. These three serines are located in the Orai1 AC8-binding sequence (amino acids 26–34). Here, we show that AC8 binding to Orai1 attenuates Orai1 serine phosphorylation in MDA-MB-231 cells, leading to enhanced Ca^2+^ entry through the channel. This statement is based on different observations. First of all, we found that AC8 and the Orai1α and Orai1β variants are overexpressed in breast cancer MDA-MB-231 and MCF7 cells as compared to non-tumoral breast epithelial cells. Secondly, Orai1α, but not Orai1β lacking the N-terminal 63 amino acids, constitutively interacts with AC8. Thirdly, while the subset of Orai1 not associated with AC8 is phosphorylated in serine residues both under resting conditions and upon stimulation with the SERCA inhibitor TG, the AC8-associated Orai1 subset is not serine-phosphorylated, thus suggesting that AC8 interaction might interfere with Orai1 phosphorylation at Ser-27, -30, and -34. Fourthly, AC8 overexpression impairs Orai1 serine phosphorylation. Lastly, AC8 expression silencing significantly reduces TG-induced Ca^2+^ entry, as well as Ca^2+^ influx mediated by co-expression of Orai1 with the STIM1 OASF region in these cells; conversely, AC8 overexpression leads to enhanced Ca^2+^ influx in cells co-expressing Orai1 and OASF, as well as TG-evoked Ca^2+^ influx.

**Figure 12:**
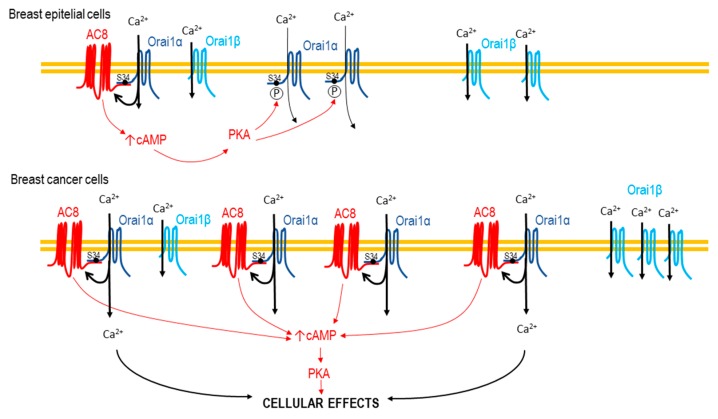
Cartoon summarizing the impairment of phosphorylation-dependent Orai1 inactivation by AC8 overexpression. In non-tumoral breast epithelial cells, Ca^2+^ influx via Orai1 activates AC8, which, in turn, results in the activation of PKA, leading to inactivation of a large subset of AC8-independent Orai1 channels. In breast cancer MDA-MB-231 cells, the greater AC8 overexpression modifies the AC8/Orai1 stoichiometry, thus preventing phosphorylation-dependent Orai1 inactivation.

A recent report by Zhang and coworkers showed an elegant functional interaction between Orai1 and AC8, where AC8 activation mediates an Orai1 inactivation mechanism driven by local cAMP and PKA activation that, in turn, phosphorylates Orai1 at Ser-34 [21]. Here, we show that binding of AC8 to Orai1, at the sequence between amino acids 26 and 34, impairs phosphorylation of Orai1 at Ser-27, Ser-30, and Ser-34, the Orai1 phosphorylation sites characterized at present [20,21,36]. Therefore, according to previous results [21], Ca^2+^ influx through the Orai1 subset associated with AC8 should be involved in the inactivation of the AC8-independent Orai1 channels. In naïve cells, with a normal AC8 expression, AC8 is expected to promote inactivation of a large subset of Orai1 channels; however, in breast cancer MDA-MB-231 cells, which predominantly overexpress AC8 over Orai1, the AC8/Orai1 stoichiometry is shifted in favor of AC8 and, subsequently, the subset of AC8-independent Orai1 is expected to be reduced, and the AC8-induced Orai1 inactivation is, thus, impaired (Figure 12).

We further explored the functional relevance of the AC8–Orai1 interaction in MDA-MB-231 cells. In breast cancer cells, Orai1 was reported to play an important role in migration [37,38] and the present study provides evidence for a role of AC8 supporting breast cancer cell migration. Silencing AC8 expression attenuates migration of resting and agonist stimulated MDA-MB-231 cells, while the same experimental maneuver was without effect in the non-tumoral breast epithelial MCF10A cell line, an observation that is likely attributed to the low AC8 expression in this cell line. The role of AC8 in cell migration is not specific to TNBC cells, as similar results were observed in the luminal breast cancer MCF7 cell line, which exhibits some common features with the MDA-MB-231 cell line, such as AC8 and Orai1 overexpression. While the role of AC8 in cell migration might be attributed to impairment of Orai1 inactivation and, thus, the support of Ca^2+^ influx, we cannot rule out the possibility that cAMP generation by AC8 also plays a role in breast cancer cell migration, as PKA activation in MDA-MB-231 and MCF7 cells by the cAMP analogue 8-bromo-cAMP per se enhances cell migration and, conversely, pharmacological PKA inhibition attenuates it. 

Finally, we investigated the mechanism underlying the role of AC8 in MDA-MB-231 cell migration. Focal adhesion turnover is essential for cell migration, and the cytosolic tyrosine kinase FAK plays a central role in the dynamics of focal adhesions [39]. Hence, we assessed whether the role of AC8 in cell migration might be attributed to the regulation of FAK phosphorylation at Tyr-397, which is used as an indicator of FAK activation [40,41]. Our results indicate that FAK phosphorylation at Tyr-397 is strongly dependent on AC8 expression, thus suggesting that FAK activation might underlie the participation of AC8 in MDA-MB-231 cell migration. 

We further observed that AC8 plays a relevant role in TNBC cell proliferation, another cellular function regulated by SOCE in breast cancer cells [3].

## 4. Materials and Methods 

### 4.1. Reagents

Fura-2 acetoxymethyl ester (fura-2/AM) was from Molecular Probes (Leiden, The Netherlands). TG, rabbit polyclonal anti-Orai1 antibody (catalog number O8264, epitope: amino acids 288–301 of human Orai1), rabbit polyclonal anti-β-actin antibody (catalog number A2066, epitope: amino acids 365–375 of human β-actin), KT5720, 8-bromoadenosine 3’-5’-cyclic monophosphate sodium, BAPTA (1,2-Bis(2-aminophenoxy)ethane-N,N,N′,N′-tetraacetic acid tetrakis(acetoxymethyl ester)), EGTA (ethylene glycol-bis(2-aminoethylether)-N,N,N′,N′-tetraacetic acid), HEPES (4-(2-Hydroxyethyl)piperazine-1-ethanesulfonic acid), EDTA (ethylenedinitrilotetraacetic acid) and bovine serum albumin (BSA) were from Sigma (St Louis, MO, USA). Rabbit monoclonal anti-FAK antibody (catalog number ab40794, epitope: within amino acids 700–800 of human FAK) and rabbit monoclonal anti-FAK (phospho Y-397) antibody (catalog number ab81298) were from Abcam (Cambridge, UK). Horseradish peroxidase-conjugated goat anti-mouse immunoglobulin G (IgG) antibody and goat anti-rabbit IgG antibody were from Jackson laboratories (West Grove, PA, USA). Clean-Blot™ IP Detection Reagent, carbamylcholine chloride (carbachol), rabbit polyclonal anti-adenylate cyclase 8 antibody (catalog number PA5-72589, epitope: amino acids 946–972 of human adenylate cyclase 8), SuperSignal® West Dura extended duration substrate reagent, Silencer® Adenylyl cyclase 8 pre-designed siRNAs (Ids#: 119586 and 119587), and Silencer® Select Negative Control siRNA were from ThermoFisher Scientific (Waltham, MA, USA). PNGase F from *Elizabethkingia miricola* was from Promega Corporation (Madison, WI, USA) DharmaFECT kb transfection reagent was from Dharmacon Inc (Lafayette, CO, USA). Protein A agarose was from Merck-Millipore (Burlington, MA, USA). Plasmids used were kindly provided by Christoph Romanin (YFP-Orai1 and YFP-OASF; University of Linz, Linz, Austria), Michelle Halls (YFP-adenylyl cyclase 8; Monash University, Australia), and Agustin Guerrero (Flag-Orai1-S27A/S30A and Flag-Orai1-S27D/S30D; CINVESTAV, Mexico). G-GECO1.2-Orai1 was a gift from Michael Cahalan (Addgene plasmid #73562; http://n2t.net/addgene:73562; Research Resource Identifier: Addgene_73562). Fura-FFP18/AM was from Santa Cruz Biotechnology (Dallas, TX, USA). All other reagents were of analytical grade. 

### 4.2. Cell Culture and Transfection

The MCF10A cell line was provided by Dr. Potier-Cartereau (Université François Rabelais Tours, France). MCF7 and MDA-MB-231 cell lines were obtained from American Type Culture Collection (Manassas, VA, USA). Hs578T cells were provided by Dr. Benitez (CNIO, Madrid, Spain). Cells were cultured up to 20–25 passages at 37 °C with 5% CO_2_ in Dulbecco’s Modified Eagle Medium (DMEM)-F12 (MCF10A) or DMEM (MCF7 and MDA-MB-231), supplemented with 10% (*v*/*v*) horse or fetal bovine serum, respectively, and 100 U/mL penicillin and streptomycin, as described previously [3]. For Western blotting and immunoprecipitation assays, cells (2 × 10^6^) were plated in 75-cm^2^ flasks and cultured for 48–72 h, while, for calcium imaging, wound healing assay, and confocal determination of G-GECO1.2 fluorescence assays, cells (2 × 10^5^ to 2.5 × 10^5^) were seeded in a 35-mm six-well multidish.

MDA-MB-231 cells were transfected with expression plasmids for YFP-Orai1, YFP-AC8, YFP-OASF, Flag-Orai1-S27A/S30A, and Flag-Orai1-S27D/S30D or scramble plasmid using DharmaFECT kb transfection reagent. Plasmids were used at 1 µg/mL. AC8 siRNAs and negative control siRNA were also transfected into cell using DharmaFECT kb transfection reagent. siRNAs were used for silencing experiments at 1 µg/mL.

### 4.3. Measurement of Cytosolic Free-Calcium Concentration ([Ca^2+^]_c_)

Cells were loaded with fura-2 by incubation with 2 μM fura 2/AM for 30 min at 37 °C as described previously [42]. Coverslips with cultured cells were mounted on a perfusion chamber and placed on the stage of an epifluorescence inverted microscope (Nikon Eclipse Ti2, Amsterdam, The Netherlands) with an image acquisition and analysis system for videomicroscopy (NIS-Elements Imaging Software, Nikon, Amsterdam, The Netherlands). Cells were continuously superfused at room temperature with HEPES-buffered saline (HBS) containing (in mM) 125 NaCl, 5 KCl, 1 MgCl_2_, 5 glucose, and 25 HEPES, pH 7.4, supplemented with 0.1% (*w*/*v*) BSA. Cells were examined at 40× magnification (Nikon CFI S FLUOR 40× Oil, Amsterdam, The Netherlands) and were alternatively excited with light from a xenon lamp passed through a high-speed monochromator Optoscan ELE 450 (Cairn Research; Faversham, UK) at 340/380 nm. Fluorescence emission at 505 nm was detected using a cooled digital sCMOS camera Zyla 4.2 (Andor; Belfast, UK) and recorded using NIS-Elements AR software (Nikon; Tokyo, Japan). Fluorescence ratio (F340/F380) was calculated pixel by pixel, and the data were presented as ΔF_340_/F_380_, as previously described [13,43,44]. TG-evoked Ca^2+^ release and Ca^2+^ mobilization, as well as constitutive Ca^2+^ influx in cells co-expressing YFP-Orai1 and YFP-OASF, were estimated as the area under the curve (AUC) measured as the integral of the rise in fura-2 fluorescence ratio for 2.5 min after the addition of TG in the absence or presence of extracellular Ca^2+^, respectively, taking a sample every second. To compare the rate of increase in fura-2 fluorescence between different treatments we used the constant of the exponential increase. Traces were fitted to the equation: *y* = A(1 − e^−K1T^ ) e^−K2T^, where K_1_ is the constant of the exponential increase. 

### 4.4. Measurement of Near-Plasma-Membrane Free-Calcium Concentration 

Cells were incubated with 5 μM fura-FFP18/AM for 2 h at 37 °C as described previously [45]. Coverslips with cultured cells were mounted on a perfusion chamber and placed on the stage of an epifluorescence inverted microscope (Nikon Eclipse Ti2, Amsterdam, The Netherlands) with an image acquisition and analysis system for videomicroscopy (NIS-Elements Imaging Software, Nikon). Cells were continuously superfused with HBS supplemented with 0.1% (*w*/*v*) BSA at room temperature and were examined at 40× magnification (Nikon CFI S FLUOR 40× Oil, Amsterdam, The Netherlands). Cells were alternatively excited with light from a xenon lamp passed through a high-speed monochromator Optoscan ELE 450 (Cairn Research; Faversham, UK) at 335 and 364 nm, and fluorescence emission, at 490 and 502 nm, respectively, was detected using a cooled digital sCMOS camera Zyla 4.2 (Andor; Belfast, UK) and recorded using NIS-Elements AR software (Nikon, Tokyo, Japan). Fluorescence ratio (F335/F364) was calculated pixel by pixel, and the data were presented as ΔF_335_/F_364_. TG-evoked Ca^2+^ entry was measured as the integral of the rise in fura-FFP18 fluorescence ratio for 3 min after the addition of extracellular Ca^2+^ taking a sample every second (AUC). To compare the rate of increase in fura-FFP18 fluorescence between different treatments, traces were fitted to the equation mentioned in Section 4.3.

### 4.5. Immunoprecipitation and Western Blotting

The immunoprecipitation and Western blotting were performed as described previously [46]. Briefly, 500-µL aliquots of cell suspension (4 × 10^6^ cell/mL) were lysed with an equal volume of ice-cold 2× NP-40 buffer, pH 8, containing 274 mM NaCl, 40 mM Tris, 4 mM EDTA, 20% glycerol, 2% nonidet P-40, 2 mM Na_3_VO_4_, and complete EDTA-free protease inhibitor tablets. Aliquots of cell lysates (1 mL) were immunoprecipitated by incubation with 2 µg of anti-Orai1 or anti-AC8 antibody and 25 µL of protein A agarose overnight at 4 °C on a rocking platform. The soft elution of proteins bound to the protein A agarose was performed according to the protocol described by Antrobus and Borner [47]. The immunoprecipitates were resolved by 10% SDS-PAGE, and separated proteins were electrophoretically transferred onto nitrocellulose membranes for subsequent probing. Blots were incubated overnight with 10% (*w*/*v*) BSA in Tris-buffered saline with 0.1% Tween-20 (TBST) to block residual protein binding sites. Immunodetection of Orai1 and β-actin was achieved by incubation for 1 h with anti-Orai1 antibody diluted 1:500 in TBST or 1 h with anti-β-actin antibody diluted 1:2000 in TBST, respectively. AC8, p-FAK, and FAK were achieved by incubation overnight with anti-AC8, anti-p-FAK (phospho Y-397) and anti-FAK antibody diluted 1:500 in TBST, respectively. The primary antibody was removed, and blots were washed six times for 5 min each with TBST. To detect the primary antibody, blots were incubated for 1 h with horseradish peroxidase-conjugated goat anti-mouse IgG antibody, horseradish peroxidase-conjugated goat anti-rabbit IgG antibody diluted 1:10000 in TBST, or Clean-Blot™ IP Detection Reagent diluted 1:250 in TBST, and then exposed to enhanced chemiluminiscence reagents for 5 min. The density of bands was measured using a C-DiGit Chemiluminescent Western Blot Scanner (LI-COR Biosciences, Lincoln, NE, USA). Data were normalized to the amount of protein recovered by the antibody used for the immunoprecipitation.

### 4.6. Wound Healing Assay

The wound healing assay was performed as described previously [3]. MDA-MB-231 cells were seeded in a 35-mm six-well multidish to obtain confluence after 24 h. Next, cells were cultured in medium supplemented with 1% serum, and a wound was created using a sterile 200-µL plastic pipette tip. Photographs were taken immediately or at the times indicated using an inverted microscope Nikon Eclipse TS100 (Tokyo, Japan). Migration of cells was quantitated using Fiji ImageJ (NIH; Bethesda, MD, USA).

### 4.7. Confocal Determination of G-GEC01.2 Fluorescence

G-GECO1.2-Orai1 transfected MDA-MB-231 cells were seeded on coverslips and mounted on a perfusion chamber and placed on the stage of an epifluorescence inverted microscope Nikon Eclipse Ti (Tokio, Japan) with an image acquisition and analysis system for videomicroscopy NIS-Elements Imaging Software (Nikon, Amsterdam, The Netherlands). Cells were continuously superfused with HBS supplemented with 0.1% (*w*/*v*) BSA at room temperature. Cells were examined at 60× magnification and excited using a confocal laser-scanning system (Melles-Griot, IDEX Health & Science, Wallingford, CT, USA) at 488 nm. Fluorescence emission at 515 nm was detected and recorded using NIS-Elements AR software (Nikon). GECO fluorescence was determined (a) before the addition of TG (resting) in the absence of extracellular Ca^2+^ (100 µM EGTA added), (b) 30 s after the addition of 1 µM TG in the absence of extracellular Ca^2+^, and (c) 30 s after the addition of 1 mM CaCl_2_ to the extracellular medium. Images were analyzed using ImageJ software (NIH, Bethesda, MD, USA).

### 4.8. Determination of Cell Proliferation

To determine cell proliferation, cells were seeded at a concentration of 5 × 10^3^/well into 96-well plates and, after 0, 24, and 48 h, cell proliferation was assessed using a specific cell proliferation assay kit based on the measurement of BrdU incorporation during DNA synthesis according to the manufacturer’s instructions (BioVision, Milpitas, CA, USA). Absorbance in samples was measured using a plate reader (Epoch, Biotek, Swindon, UK) at 450 nm, presented as arbitrary units.

### 4.9. Statistical Analysis

Analysis of statistical significance was performed using the Kruskal–Wallis test combined with Dunn´s post hoc test (or one-way analysis of variance combined with Tukey post hoc test for the analysis of Ca^2+^ determinations) (GraphPad Prism Windows 5.04, San Diego, CA, USA). For comparison between two groups, the Mann–Whitney U test was used. A *p*-value <0.05 was considered to be statistically significant. 

## 5. Conclusions 

In conclusion, our results provide strong evidence for the impairment of phosphorylation-dependent Orai1 inactivation by AC8 overexpression in MDA-MB-231 cells, a mechanism that enhances Ca^2+^ influx, breast cancer cell migration (by supporting FAK activation), and cell proliferation. These findings suggest that AC8 might be a good candidate for the development of anti-tumoral strategies in breast cancer.

## Supplementary Materials

The following are available online at https://www.mdpi.com/2072-6694/11/11/1624/s1: Figure S1: Ca^2+^ mobilization near the plasma membrane in MDA-MB-231 breast cancer cells; Figure S2: Role of the cAMP–PKA pathway in MCF7 cell migration; Figure S3: Role of AC8 in MCF7 cell migration; Figure S4: Role of AC8 in MCF10A cell migration.
