# Supplementary material for: Adenylyl Cyclase Type 8 Overexpression Impairs Phosphorylation-Dependent Orai1 Inactivation and Promotes Migration in MDA-MB-231 Breast Cancer Cells
Source: Cancers (Basel). 2019 Oct 23;11(11):1624. doi: 10.3390/cancers11111624 (PMC6893434; doi:10.3390/cancers11111624)
Supplement: Supplementary file 1 [file cancers-11-01624-s001.zip › cancers-625953 - Western blot figures-final.pdf]

Figure 1a

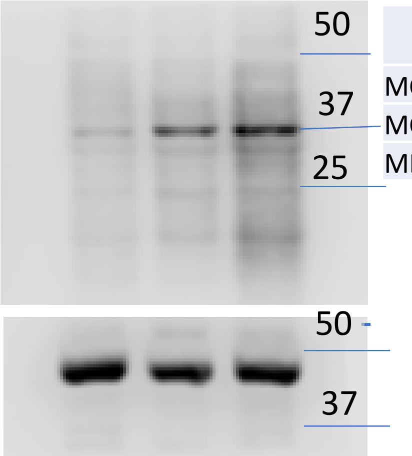

	ORAI1	ACTIN	RATIO
MCF10A	11636024	69989917,5	0,16625286
MCF7	27272258	62608750,5	0,43559818
MDAMB231	46942371	71336145	0,65804468

Figure 1c

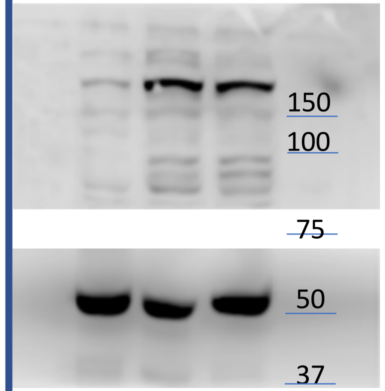

	AC8	ACTIN	RATIO
MCF10A	1159891	19607714	0,05915483
MCF 7	6825518	16913250	0,4035604
MDA MB 231	6636397	18458522	0,35953025

Figure 1e

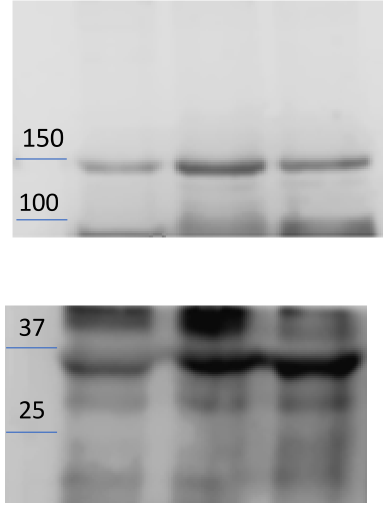

	AC8	Orai1	Ratio
MCF 10A	3466793,33	11630187	0,29808578
MCF7	9606248	16553066	0,58033044
MDA MB 231	7494511,33	13984459	0,53591714

Figure 1g

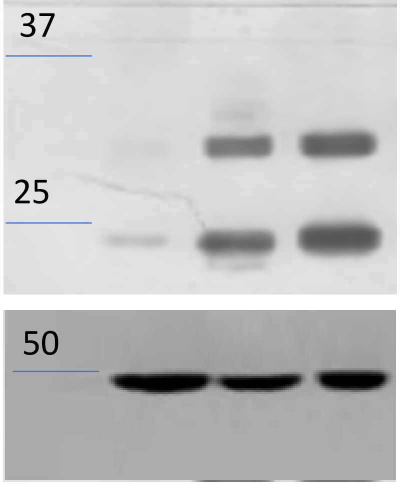

	Orai1α	Orai1β	actin	Orai1α/actin	Orai1β/actin	ratio α/β
MCF 10A	118924,5	275813,5	5339598	0,022272182	0,051654357	0,43117723
MCF7	1443910	1918945,5	4264477	0,338590172	0,449983785	0,75244972
MDA MB 231	2700663	3504516,5	4083184	0,661411046	0,858280327	0,77062357

Figure 1j

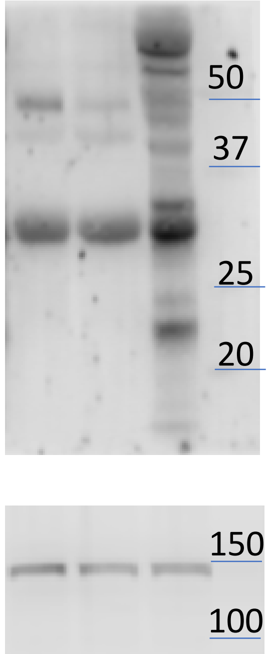

Figure 2a

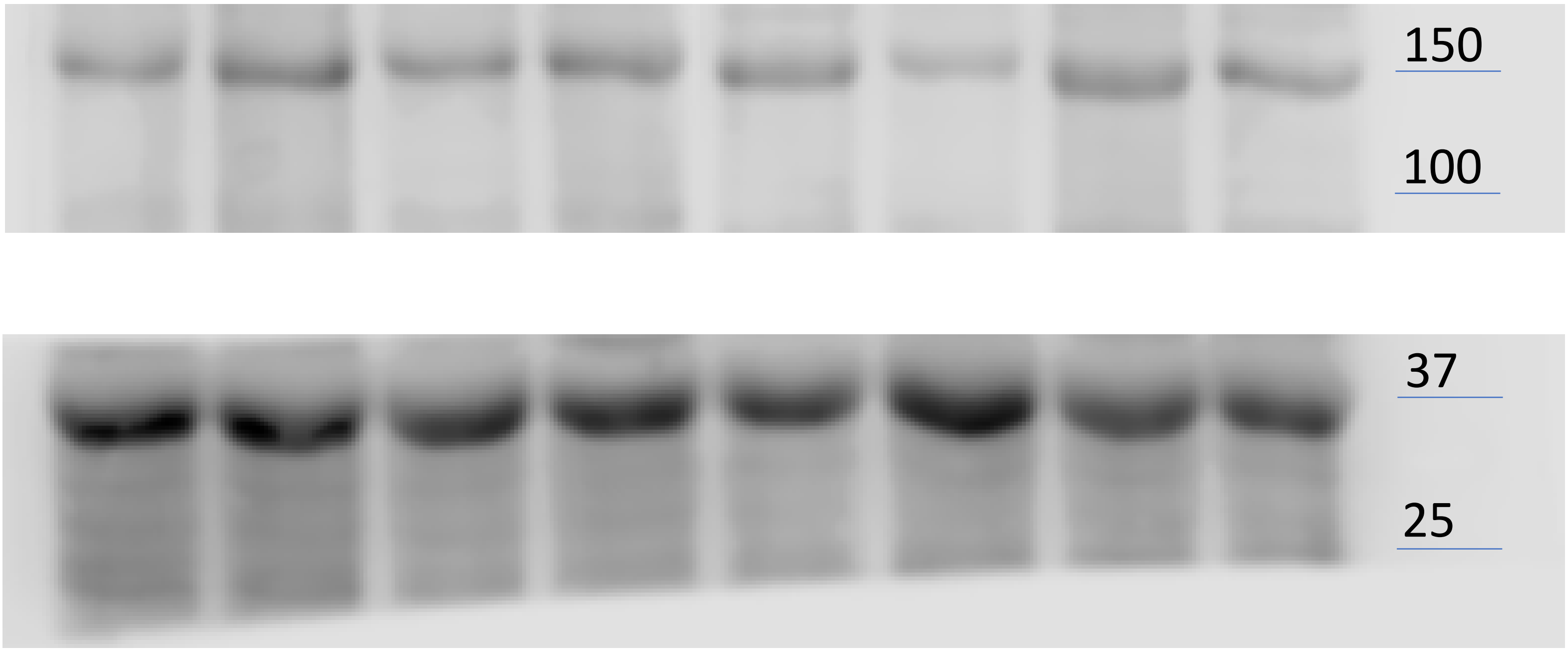

		AC8	Orai1	Ratio AC8
	C	2.391.864	5.329.891	0,44876416
	TG	3.627.816	6.980.296	0,51972242
	C (BAPTA)	2.663.734	5.916.719	0,45020466
CALCIO	TG (BAPTA)	3.118.205	6.605.083	0,47209175
	C	2.403.671	5.468.376	0,43955849
	TG	1.392.923	7.698.912	0,1809246
	C (BAPTA)	2.736.372	5.876.447	0,4656508
EGTA	TG (BAPTA)	1.981.295	5.555.598	0,35663041

Figure 3

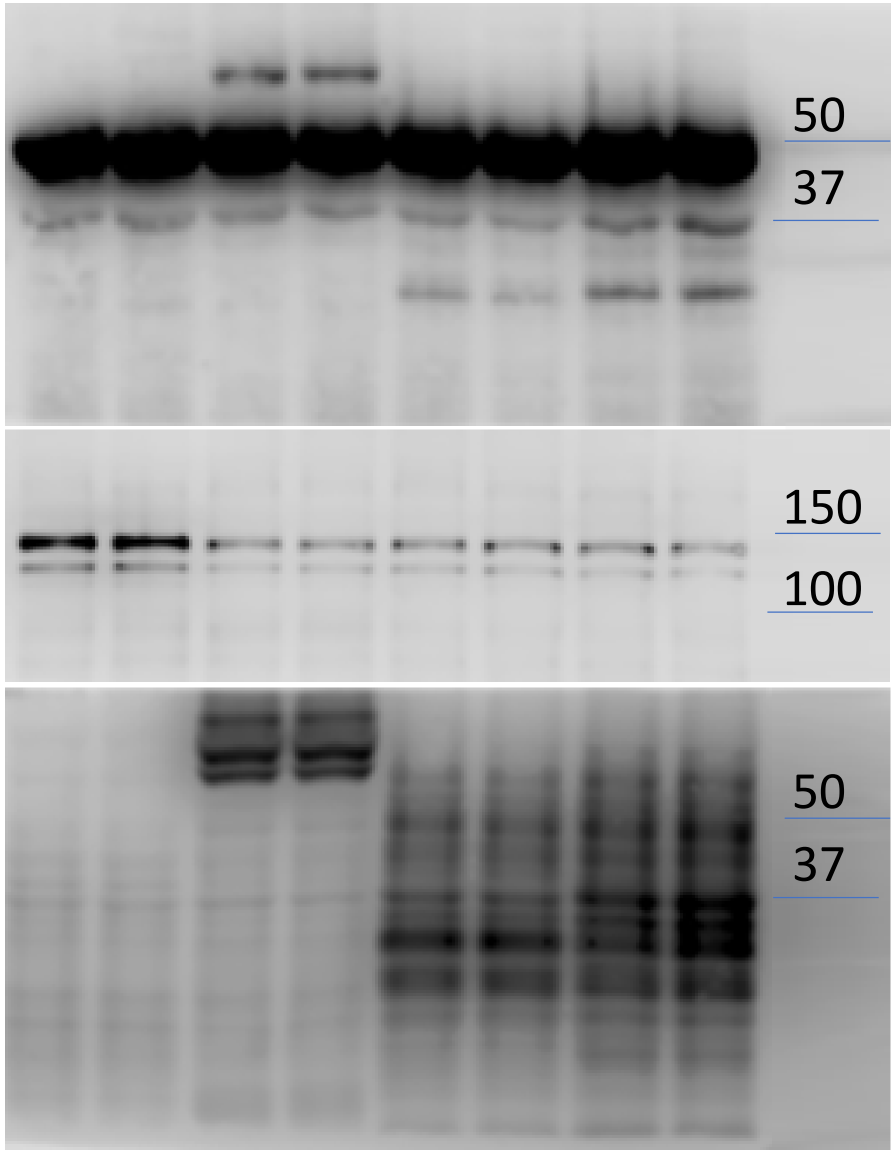

		AC8	Orai1 native	Orai1 mutants	orai1-YFP
Mock	C	56386459	29951409		
	TG	57123288	35709924		
Orai1YFP	C	28167974	27145146		51660513
	TG	24624338	23639388		53364785
Orai1-S27AS30A	C	26366388	28774510	15825217	
	TG	27703853	30159338	10746874	
Orai1-S27DS30D	C	28692146	49443137	27950087	
	TG	19442510	48859430	29551945	

Figure 4

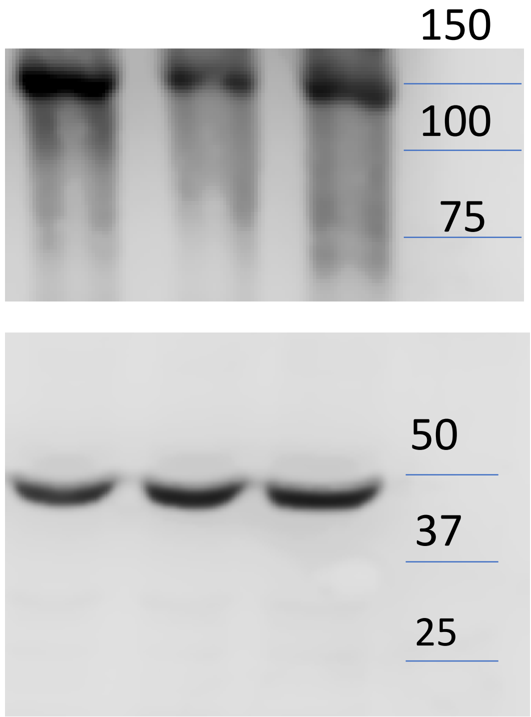

	AC8	Actin	ac8/actin
Sc	2348583	5607811	0,41880566
#1	1156316	64198761	0,0180115
#2	1261721	6912296	0,18253284

Figure 6

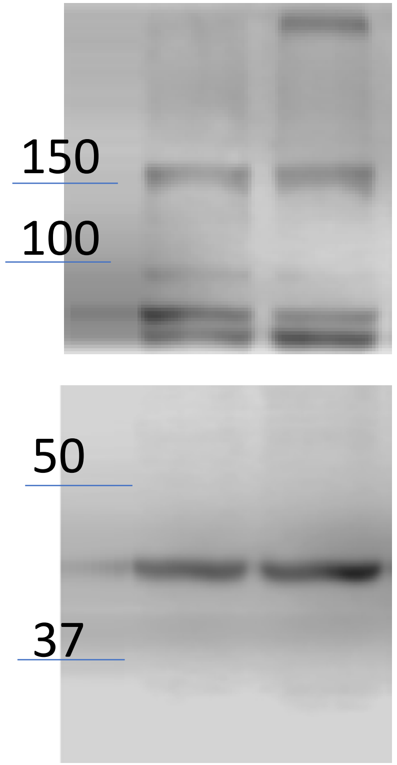

	AC8 native	AC8 YFP	actin
mock	53192434		77026752
SOB AC8	66077614	64981312	84799851

Figure 7a

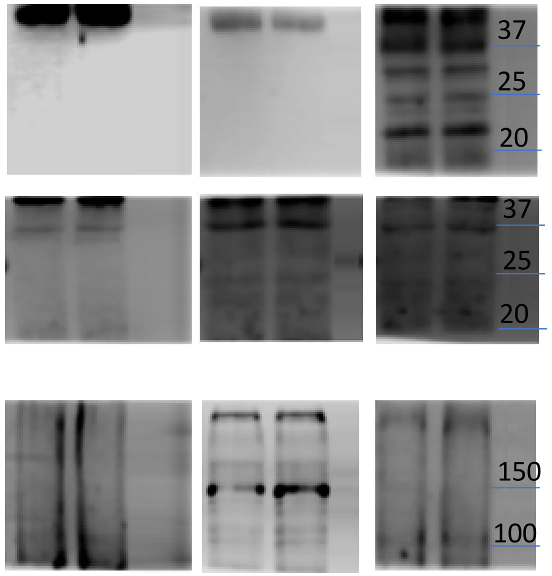

Figure 7b

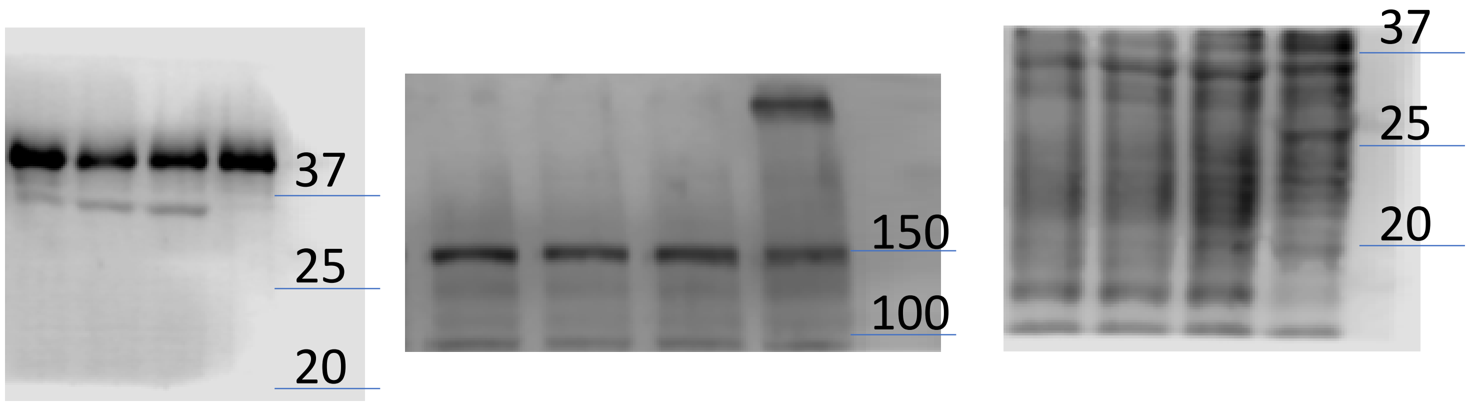

	Phospho Orai1	Orai1	Phospho Orai1/Orai1	AC8 native	AC8-YFP
mock	722134	1491317,5	0,484225525	2482841	
SiAC8 #1	1581205	2789602,5	0,5668209	2241841	
SiAC8#2	2350497	3109475	0,755914423	2554912	
AC8-YFP	132920	2117175	0,062781773	1730497	3878004

Figure 10

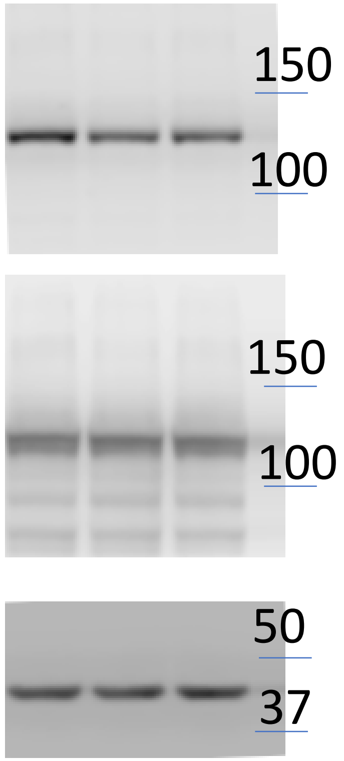

	pFAK	FAK	pFAK/FAK	actin
mock	4983761	7313108,4	0,68148327	2858012
SiAC8#1	3768104	7022289,3	0,53659196	2840305
SIAC8#2	3823347	7012138,9	0,5452469	3025891

Figure 11

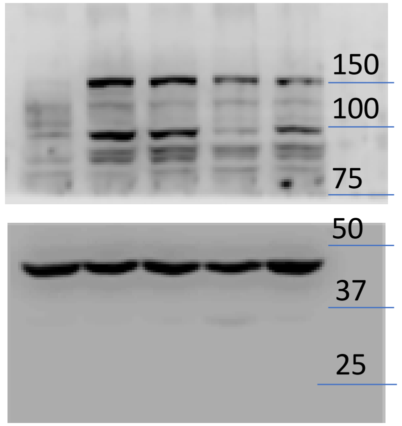

	AC8	actin	AC8/actin
MCF10A	10890761	79193433	0,13752101
MCF7	50991874	87594750,5	0,5821339
MDAMB231	44842874	85368720	0,5252846
BT20	30393238	75602872,5	0,40201168
Hs578T	26783016	89056315,5	0,30074247

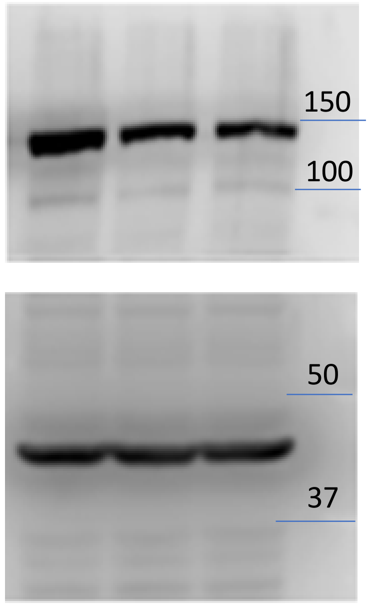

	AC8	actin	Ac8/actin
control	54.301.489	110671144	0.49065625
SiAC8 #1	38.895.267	107282250	0.36255081
SiAC8 #2	37.053.137	109616274	0.33802588
