# Supplementary material for: Adenylyl Cyclase Type 8 Overexpression Impairs Phosphorylation-Dependent Orai1 Inactivation and Promotes Migration in MDA-MB-231 Breast Cancer Cells
Source: Cancers (Basel). 2019 Oct 23;11(11):1624. doi: 10.3390/cancers11111624 (PMC6893434; doi:10.3390/cancers11111624)
Supplement: Supplementary file 1 [file cancers-11-01624-s001.zip › cancers-625953-supplement-final.pdf]

Supplementary Material

Adenylyl cyclase type 8 overexpression impairs phosphorylation-dependent Orai1 inactivation and promotes migration in MDA-MB-231 breast cancer cells

Jose Sanchez-Collado, Jose J. Lopez, Isaac Jardin, Pedro J. Camello, Debora Falcon, Sergio Regodon, Gines M. Salido, Tarik Smani and Juan A. Rosado

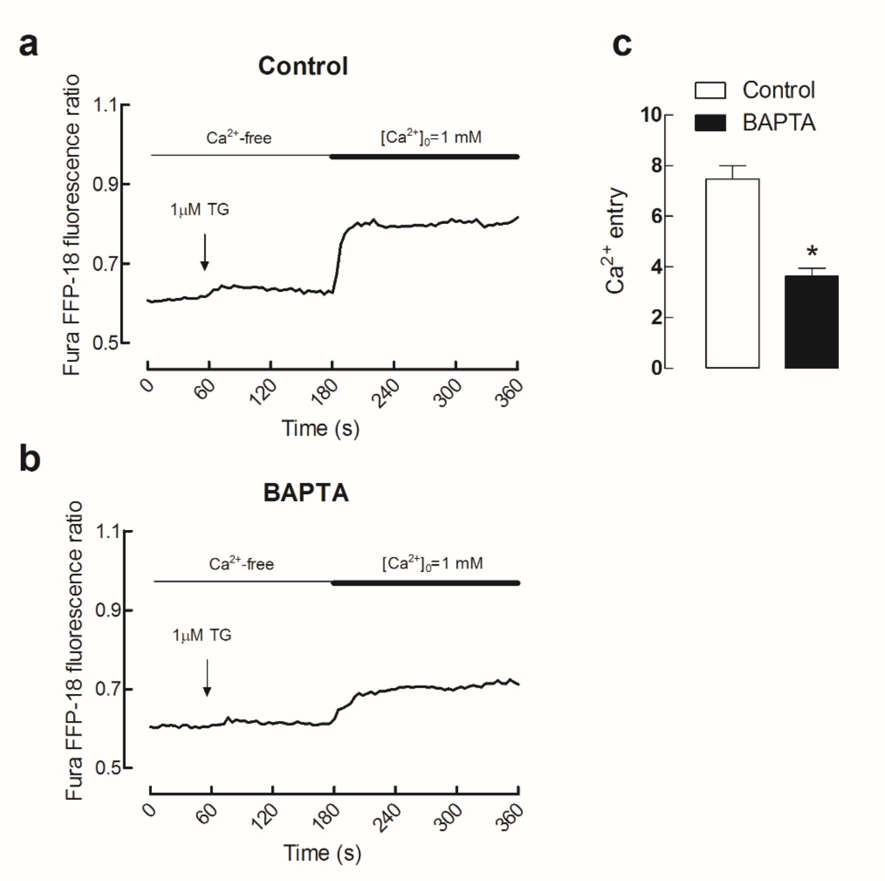

Figure S1. Ca^{2+} mobilization near the plasma membrane in MDA-MB-231 breast cancer cells. MDA-MB-231 cells were loaded with dimethyl-BAPTA (**b**) or left untreated (**a**). Cells, loaded with fura FFP-18, were perfused with a Ca^{2+}-free medium (100 μM EGTA added) and stimulated with 1 μM TG followed by reintroduction of external Ca^{2+} (final concentration 1 mM) to initiate Ca^{2+} entry. Traces are representative of 40 cells/day/3–5 days. (**c**) Bar graphs represent Ca^{2+} mobilization near the plasma membrane upon reintroduction of external Ca^{2+} in BAPTA-loaded or untreated (Control) cells, estimated as described in Material and Methods.

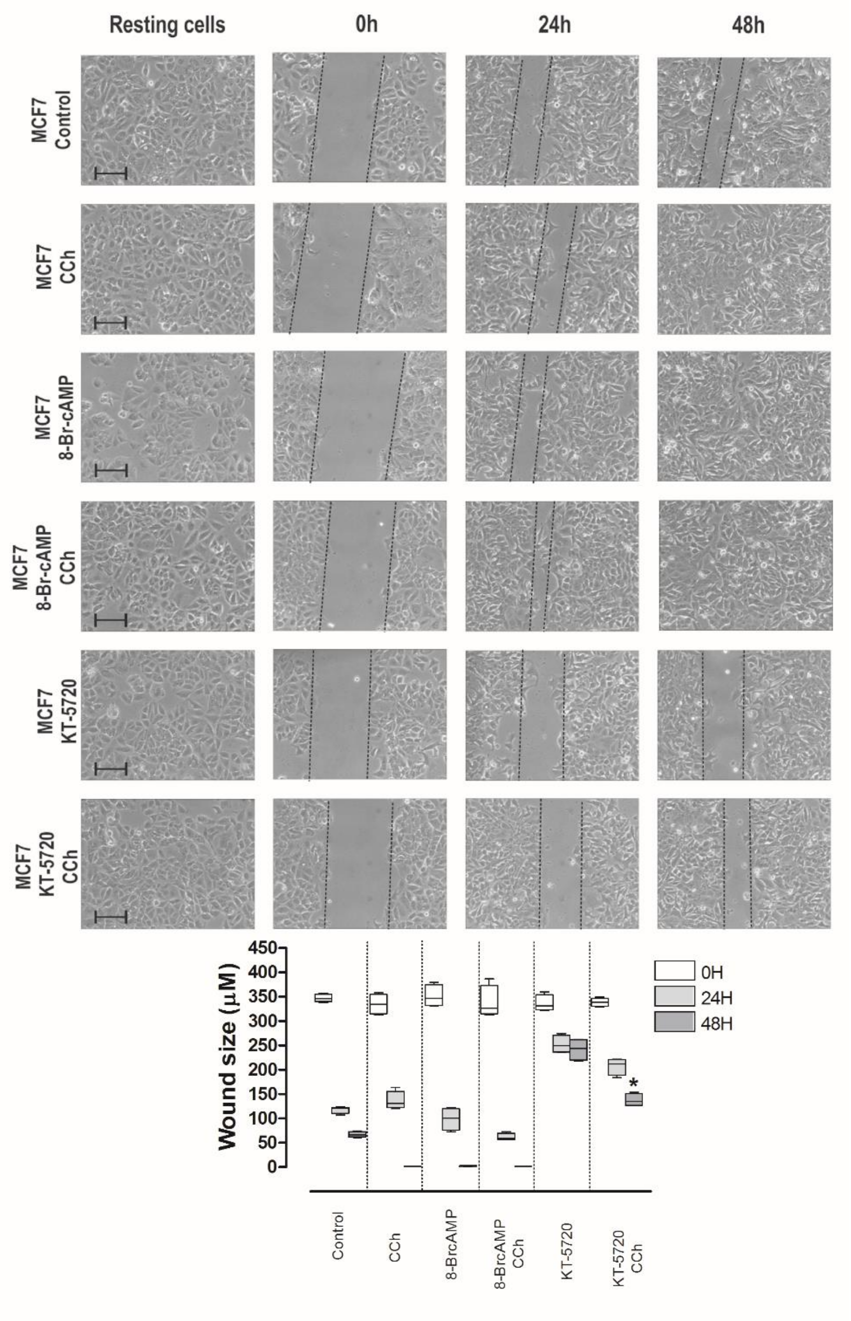

Figure S2. Role of the cAMP-PKA pathway in MCF7 cell migration. MCF7 cells were stimulated with 10 μM CCh or the vehicle in the absence or presence of 8-Br-cAMP (300 μM) or KT-5720 (1 μM), and subjected to wound healing assay as described in Methods. Images were acquired at 0, 24 and 48 h from the beginning of the assay. The dotted lines define the areas lacking cells. The bars represent 100 μm. The box plot represents the wound size, in micrometers, at the different conditions (n = 6). * $p < 0.05$ compared to the corresponding time in CCh-treated cells.

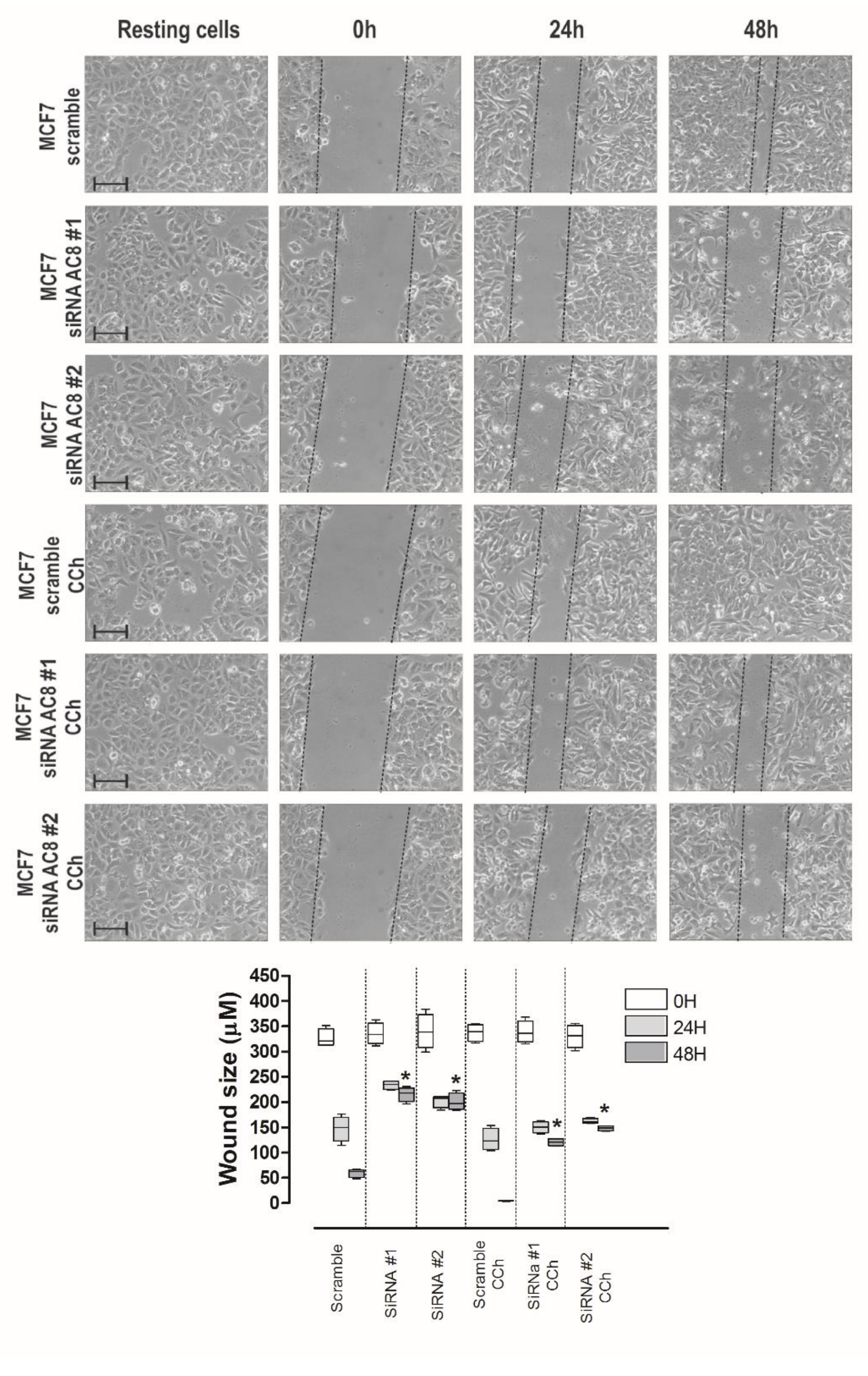

Figure S3. Role of AC8 in MCF7 cell migration. MCF7 cells were transfected with siAC8#1, siAC8#2 or scramble plasmid, as indicated. Forty-eight hours after transfection cells were stimulated with 10 µM CCh or the vehicle and subjected to wound healing assay as described in Methods. Images were acquired at 0, 24 and 48 h from the beginning of the assay. The dotted lines define the areas lacking cells. The bars represent 100 µm. The box plot represents the wound size, in micrometers, at the different conditions (n = 6). * $p < 0.05$ compared to the corresponding time in cells transfected with scramble plasmid.

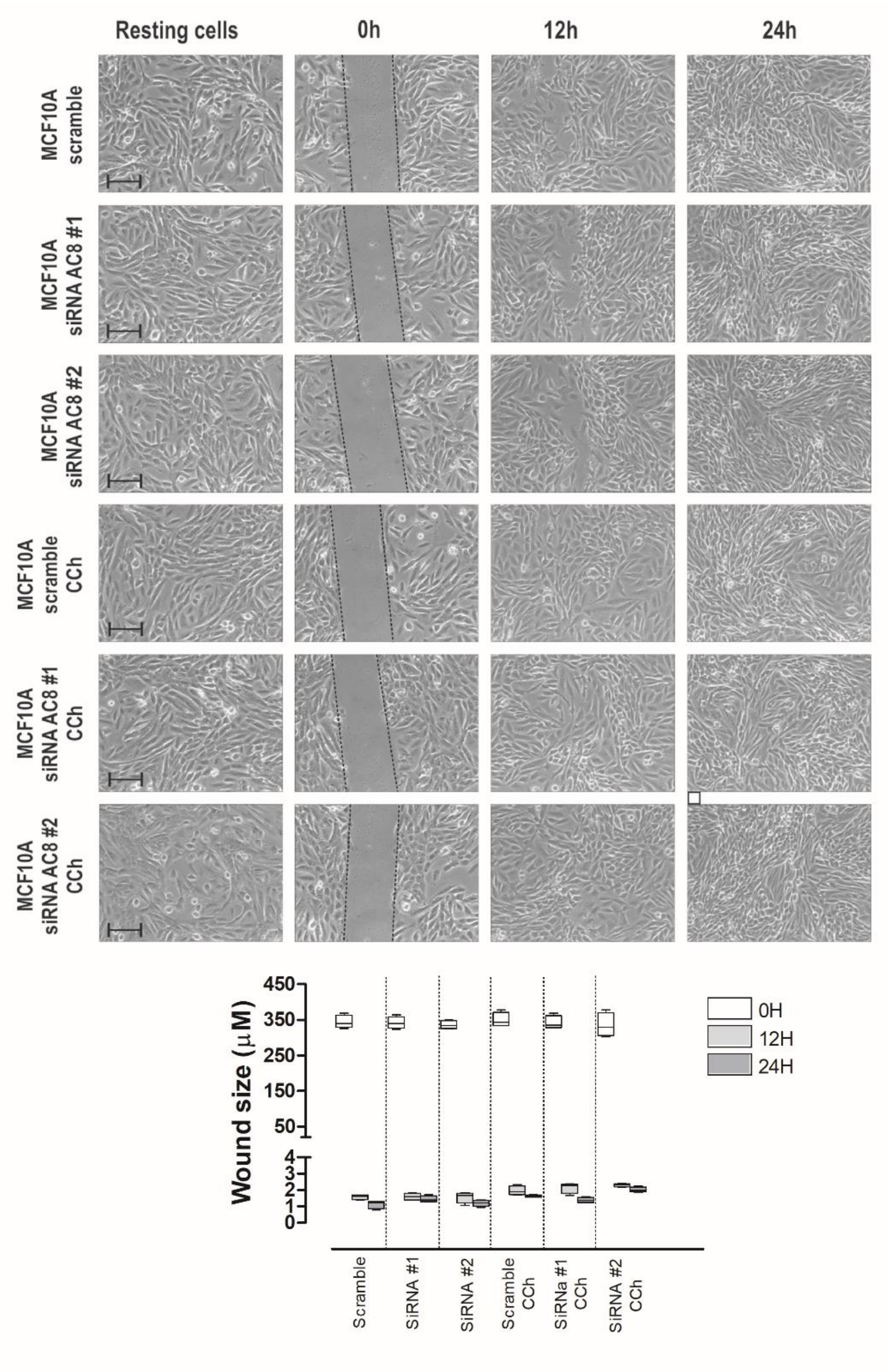

Figure S4. Role of AC8 in MCF10A cell migration. MCF10A cells were transfected with siAC8#1, siAC8#2 or scramble plasmid, as indicated. Forty-eight hours after transfection cells were stimulated with 10 μM CCh or the vehicle and subjected to wound healing assay as described in Methods. Images were acquired at 0, 12 and 24 h from the beginning of the assay. The dotted lines define the areas lacking cells. The bars represent 100 μm. The box plot represents the wound size, in micrometers, at the different conditions (n = 6).
